# Psychological antecedents of excess gestational weight gain: a systematic review

**DOI:** 10.1186/s12884-015-0535-y

**Published:** 2015-05-02

**Authors:** Mufiza Zia Kapadia, Anca Gaston, Sherry Van Blyderveen, Louis Schmidt, Joseph Beyene, Helen McDonald, Sarah D McDonald

**Affiliations:** Department of Obstetrics and Gynecology, McMaster University, Hamilton, Canada; School of Kinesiology, University of Western Ontario, London, Ontario Canada; New Leaf Psychology, Milton, Canada; Department of Psychology, Neuroscience & Behavior, McMaster University, Hamilton, Canada; Department Clinical Epidemiology and Biostatistics, McMaster University, Hamilton, Canada; Midwifery Education Program, Department of Family Medicine, McMaster University, Hamilton, Canada; Division of Maternal-Fetal Medicine, Departments of Obstetrics & Gynecology, Radiology, and Clinical Epidemiology & Biostatistics, McMaster University, 1280 Main Street West, room 3N52B, Hamilton, Ontario L8S 4K1 Canada

**Keywords:** Psychological factors, Pregnancy, Weight gain, Systematic review

## Abstract

**Background:**

Excess gestational weight gain (GWG), which has reached epidemic proportions, is associated with adverse outcomes during pregnancy and postpartum obesity in women and children. Psychological variables represent potentially modifiable factors. Moreover, previous systematic reviews on GWG interventions have called for the need for a clearer understanding of psychological factors affecting GWG. Hence, a systematic review was conducted to summarize the relation between psychological factors and GWG.

**Methods:**

Eight databases were searched, and the guidelines on Preferred Reporting Items for Systematic Reviews and Meta-Analyses were followed. Methodological quality of the included studies was assessed using a modified Newcastle-Ottawa scale. Two assessors independently reviewed titles, abstracts and full articles, extracted data and assessed quality.

**Results:**

A total of 6198 titles and abstracts were reviewed of which 90 full text articles were retrieved. Thirty-five studies (25 cohort, eight cross-sectional and two case–control) met the inclusion criteria, assessing 26 different psychological constructs in affect, cognitions and personality. Negative affective states such as depression, anxiety and stress were not related to excess GWG. Among weight-related and dietary-related cognitions, risk factors for excess GWG included concern about weight gain, negative body image and attitude towards weight gain, inaccurate perceptions regarding weight, higher than recommended target weight gain, less knowledge about weight gain, higher levels of cognitive dietary restraint, and perceived barriers to healthy eating. Protective factors included an internal locus of control for weight gain, lower than recommended target weight gain and higher self-efficacy for healthy eating. Only one study examined the relation between personality and excess GWG.

**Conclusion:**

In this systematic review, a number of cognitive factors were identified that were associated with excess GWG. To address excess GWG, more high quality, adequately powered studies are required examining cognitions, motivation and personality factors.

**Electronic supplementary material:**

The online version of this article (doi:10.1186/s12884-015-0535-y) contains supplementary material, which is available to authorized users.

## Background

Excess gestational weight gain (GWG) i.e., weight gain above that recommended Institute of Medicine (IOM) guidelines [1] occurs in half or more of women in many populations [2-6]. Excess GWG significantly increases the risks of serious maternal pregnancy complications such as gestational diabetes mellitus, gestational hypertension and caesarean section [1]. Excess GWG is also associated with an increased risk of obesity in mothers during the postpartum [7,8]. Some, but not all women who are overweight or obese have excess GWG [9]. Neonates born to women who have excess GWG are at risk of being born large for gestational age [10,11]. Being born large increases risks to the infants’ health, both immediately after birth, (including seizure, hypoglycemia, polycythemia, and meconium aspiration [11]) and in the long term (including adiposity [12]). Thus, a cycle of obesity can be transmitted across generations.

To combat the immediate and long-term adverse outcomes associated with excess GWG, guidelines on GWG were published in 1990 by the US Institute of Medicine (IOM) [13], and updated in 2009 [1]. These guidelines were also adopted by other countries such as Canada and Finland [14]. Given that a high proportion of women gain above the recommendations, achieving a better understanding of factors related to excess GWG is a public health priority.

In contrast to biological or demographic determinants of excess GWG such as age, parity, ethnicity, or socioeconomic status, psychological variables represent potentially modifiable factors during pregnancy. Moreover, previous systematic reviews on GWG interventions have called for the need for a clearer understanding of psychological factors affecting weight gain. For example, Gardner highlighted a need to measure psychological mechanisms underpinning behavior change [15]. Kramer and colleagues suggested that whereas dietary modifications during pregnancy lowered GWG [16], interventions that focused on increasing physical activity through aerobic exercise were ineffective in reducing GWG [17]. These conclusions suggest that dietary modifications are critical in a pathway of behavioral modifications to GWG. These findings are further supported by systematic reviews of clinical trials that suggest that physical activity plus dietary interventions [18,19] are most effective in reducing GWG. One of those systematic reviews [18] found no effect on GWG with supervised physical activity *alone*. These findings further strengthen the key role of dietary interventions in reducing total GWG [20]. Skouteris and colleagues recommended that it was important to focus on the *antecedents* of psychological behavior which have previously not been targeted in interventions [21]. The authors particularly noted that psychological factors such as *affect* (feelings and emotional reactions to an occurrence [22-24]), and *cognitive factors* (thoughts and beliefs about the occurrence [22-24]) such as body image concerns, self-efficacy about making behavioral changes, and motivation might impede behavioral changes, and should therefore be targeted along with behavioral changes. Outside of pregnancy, another systematic review found one broad element of the Five Factor Model of personality, conscientiousness, was important. Moreover, this study suggested the need for future studies to examine lower level personality facets in relation to obesity prevention and treatment strategies [25]. A recent large cohort of young adults found that the effect sizes of various personality traits were on par with other well-established health risk factors such as socioeconomic status and smoking at predicting poor health in midlife [26]. Other research demonstrated that the magnitude of the effect of personality traits on mortality was similar to that of socioeconomic status [27]. Personality’s role in preventive health care was deemed so pivotal that the American Psychological Association issued a recent Press Release entitled “Personality May be Key Risk Factor in Preventive Health Care” [28].

To date, there appears to be no systematic review that has addressed the relation between these psychological antecedents of pregnancy behavior and excess GWG. As a result, little is known about the psychological protective and risk factors associated with excess GWG. Hence, the aim of this systematic review was to provide a summary of the available evidence examining psychological antecedents of excess GWG, investigating three broad psychological domains, namely, *affect*, *cognitions* (related to dietary behavior, weight gain, or physical activity) and *personality*.

## Methods

A systematic review of the literature was carried out in accordance with PRISMA (Preferred Reporting Items for Systematic Reviews and Meta-Analyses) [29] and Cochrane Handbook for Systematic Reviews of Interventions [30]. Separate, specific electronic searches were created with the aid of experienced reference librarians specializing in health sciences and psychology and conducted in the following databases from their inception (dates included wherever available in the databases) until May 3, 2013: Medline (1946 to 21 Jan 2015), EMBASE (1974 to 21 Jan 2015), PsychINFO (1806 to 21 Jan 2015), Cochrane Register, CINHAL, Web of Knowledge, Sociological Abstract, and Dissertation and Thesis (see Supporting information in Additional file 1 for the search strategies developed in the eight databases). Since it was a systematic review and did not directly involve human subjects, ethical approval was not required.

### Study inclusion criteria

#### Study design

The following study designs were included: randomised controlled trials, cross-sectional studies, case–control studies and cohort studies. Reviews, editorials and opinion articles were excluded, since they did not contain primary data. Studies published as abstracts only (e.g., conference proceedings) were excluded, since their quality could not be adequately assessed. Duplicate or secondary publications on the same exposure in the same population were excluded to avoid multiple publication bias. There were no language restrictions.

#### Participants

Studies were restricted to women who were pregnant with a singleton or if recently postpartum, had already delivered a singleton.

#### Independent variables (exposures)

Studies were included if one or more of the psychological constructs, falling under the broad headings of *affect*, *cognitions* (related to dietary behavior, weight gain, or physical activity) or *personality* were assessed as exposure variables (Table 1). (However, examination of constructs related to physical activity are labeled ‘post hoc’ for transparency’ sake, as a secondary objective, since they were added after the the initial development of the objectives and search strategy.) Studies that focused on the association between psychiatric disorders and GWG were excluded: We excluded studies that focused on a psychiatric diagnosis such as Major Depressive Disorder or a formal diagnosis of anxiety disorder, defined according to psychiatric criteria. We did this in two ways: Firstly, the Medical Subject Headings (MeSH) terms we used were designed to identify less severe forms of depression and anxiety, i.e. depressive and anxiety states of “mild to moderate” intensity. These terms we selected were in contrast to major depression or major anxiety. Secondly, when we screened the articles, we excluded ones focusing on major depression or anxiety.

**Table 1:** **Psychological concepts covered in systematic review of psychological antecedents of excess gestational weight gain** ^**$**^

**Psychological construct**	**Themes** ^$$^
**1. Affect**	Stress
	Distress
	Adaptation/coping behavior
	Depression
	Anxiety
	Mood/Affect
	Emotions/Feelings
**2. Cognitions**	
**Related to weight gain**	Self-esteem
	Self-efficacy
	Locus of control
	Body image
	Attitude
	Motivation
**Related to dietary behavior**	Eating attitudes
	Feeding behavior
	Knowledge
**Personality**	Personality traits related to Five Factor Model and Eysenck’s personality model
	Resilience
	Impulsivity

^$^Search strategies related to these psychological constructs are provided in supporting information Additional file 1.

^$$^Studies were excluded if they measured a psychiatric disorder.

#### Outcome measures

##### Primary outcomes

Studies were included if they had one or more of the following categorical outcomes of interest: total GWG in excess of the guidelines (i.e., weight gain above the recommended Institute of Medicine (IOM) guideline of 1990 [13] or 2009 [1]; rate of GWG (mean range of weight gain per week) above the IOM guidelines; or high adequacy ratio of GWG (expressed as the ratio of observed/expected total weight gain) above the IOM guidelines. *Secondary outcome:* Studies which defined GWG as a continuous outcome (as total GWG, either with or without reference a prepregnancy BMI) were included as secondary outcomes.

#### Study selection process

Two reviewers independently assessed the titles and abstracts of all identified citations (MZK and AG). Full-text articles were retrieved if either reviewer considered the citation potentially relevant with a low threshold for retrieval. The bibliographies of studies included for full-text review were also checked for additional relevant references. Inter-reviewer agreement for decision for full text review was assessed using an un-weighted kappa statistic. The final set of studies which was included in the systematic review was determined by consensus (between MZK and AG) with any disagreements resolved by the third reviewer (SDM).

#### Assessment of risk of bias

The methodological quality of the studies was assessed using a modified version of the Newcastle-Ottawa Scale [31]. The Newcastle-Ottawa Scale is comprised of three categories: ‘Selection,’ ‘Comparability,’ and ‘Outcome’ for cohort studies, and ‘Selection,’ ‘Comparability,’ and ‘Exposure’ for case control studies. Within the category of ‘Selection,’ one item was excluded for cohort studies and one item was excluded for case–control studies, namely ‘Demonstration that outcome of interest was not present at start of study’ and ‘Definition of controls: no history of disease (endpoint),’ respectively. These items were not relevant in the context of the present systematic review since our outcome of interest, GWG, could not have been present at the time of recruitment. To ensure the proper ascertainment of psychological constructs, the use of validated psychological scales was deemed to be important, hence the regular Newcastle-Ottawa Scale scoring items ‘ascertainment of exposure’ under the category of ‘Selection’ in cohort studies and ‘Exposure’ in case–control cohort studies were replaced with the following items: ‘≥50% of the tool(s) are stated /known validated’ or ‘≥50% of the tool(s) are validated, but modified.’ One point was awarded for ascertainment of exposure resulting in a maximum score of three points for ‘Selection.’

With respect to criteria related to ‘Comparability’, it was decided *a priori* that studies would receive one point if they controlled for pre-pregnancy BMI, as this is a key determinant of excess GWG [1]. Studies were given an additional point if they controlled for one or more of the following five potential confounders: age, parity, income, education or race. In evaluating the outcome, to be awarded points we required: that weight be measured objectively not self-reported, that there be sufficient duration of follow-up (we required that total GWG be evaluated at 37 weeks or beyond , the time frame which is considered ‘term’) and that there be complete or near complete (>90%) follow-up. In total, our modified Newcastle-Ottawa Scale awarded up to eight points. As there is no validation study that provides a cut off score for rating low quality studies, a cut off of less than 5 was used to consider a study as ‘low quality’ in this review. However, the studies were not excluded from the analysis in order to avoid publication bias based on their quality and because there is no validated NOS cut-off for excluding studies.

In addition, power was assessed by applying the general rule that regression models require a minimum of 10 events per predictor variable [32]. In cases where the number of variables entered into the model could not be determined, the study was categorized as ‘unable to determine power.’ For studies that performed only univariate analyses, a Bonferroni correction for multiple comparisons was applied, by dividing the *p-*value (α) of the test by the number of comparisons made in the study [33]. If the *p*-value for any given comparison was greater than the newly calculated *p*-value, then the study was classified as under-powered.

Thus, overall study quality was assessed through both the modified Newcastle-Ottawa Scale and the aforementioned power assessment, and is presented in the results. For exposures that were examined by five or more studies, we planned to assess publication bias with a funnel plot.

#### Data abstraction

To ensure consistency, an electronic data abstraction form was developed and subsequently piloted by three reviewers (MZK, AG, SDM) on one study [34]. For the remaining studies, two reviewers (MZK and AG) independently abstracted all available data. The following information was extracted from the included studies: date of publication, design, years the study was conducted, setting, population, inclusion/exclusion criteria, study outcomes, psychological scales/instruments, the timing of assessment of psychological exposure during gestation, and the results. Inconsistencies were checked and resolved through the consensus process described above.

#### Data synthesis

Data were not pooled for several reasons: Firstly, even when multiple studies examining the same exposure were available, different scales or, in some cases modified versions of the same scale, were often used. Secondly, some of the studies did not present an effect estimate, exact *p-*value, or sufficient data for it to be calculated, and instead only reported the presence and direction of an association. Thirdly, it was decided *a priori* that when available the results of a multivariate analysis would be preferentially reported. Lastly, some studies only reported effect estimates for a few items within a scale, or for subscales, and the effect estimates were not comparable across different studies.

Forest plots were generated in Revman 5.2 (Copenhagen, Denmark) to visually represent the magnitude and direction of the association of each exposure with the primary outcome. However, the pooled effect estimates were not reported in the forest plots due to the heterogeneity of the data. Instead two additional methods were used to visually represent our findings, which we have termed a pinwheel and a web plot. For the pinwheel, the center was divided into three main psychological constructs outlined in Table 1, namely affect, cognitions and personality and direction of association were shown through color coded boxes, each box representing an individual study.

## Results

**Figure 1:**
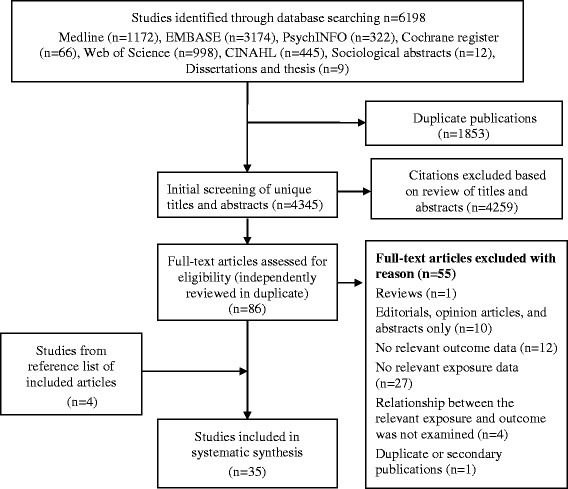
Flow diagram of study selection process in systematic review of psychological antecedents of excess gestational weight gain.

Our initial searches of the eight databases yielded 6198 records (Figure 1). After removal of duplicates (n = 1853), 4345 studies remained and were assessed based on titles and abstracts. Based on our screening criteria, 86 papers were selected for full-text review. Four additional papers were found through perusal of reference lists of full text of the included studies. A total of 35 studies [2,9,34-66] met inclusion criteria. The un-weighted κ for initial agreements on full study inclusion between two reviewers was 0.973. Twenty seven studies [2,9,35-38,40-43,45,46,48-59,61-63] reported our primary outcome of interest i.e., excess GWG as a categorical outcome.

### Study characteristics

Baseline information on 25 cohort studies, eight cross-sectional studies and two case–control studies are presented in Tables 2 and 3. None of the included papers were in the format of RCTs (although a few secondary analyses of RCTs were included, the data in these instances were in the format of cohort studies). The majority of studies were from the United States (n = 26) and the remaining studies were from the United Kingdom [38], Canada [54,61], Australia [42,62,64], Japan [47], China [65] and Iran [35]. All of the included studies were in English. A total of 18,828 women were included, and study sizes ranged from 46 to 4528 participants. Study populations included women from most often drawn from the general population, although a few focused on specific populations (particular ethnic groups:2 studies, adolescents: 2 studies and low income populations:2 studies). There were several publications from the same cohort but they examined different psychological exposures with excess GWG: four studies examined the Pregnancy, Infection and Nutrition (PIN) cohort [2,43,46,48], two studies examined Rochester Study of Adolescent Pregnancy cohort [45,52] and two studies examined data collected as part of the ongoing Pregnancy Risk Assessment Monitoring System (PRAMS) [56,58]. The timing of assessments of exposure varied widely between the studies. Most studies assessed exposures in second trimester [2,34,36,38,39,41,42,45-50,52-55,59,60,62,64,66], however, some studies assessed exposures in third trimester [9,35,37,40,43,44,61,62,65] or retrospectively during the immediate postpartum period [51,56-58]. The psychological scales used in the ascertainment of exposure are described in Additional file 1: Table S1.

**Table 2:** **Characteristics of included cohort studies in systematic review of psychological antecedents of excess gestational weight gain**

**Author, year (** ***years study span*** **)**	**Sample size**	**Setting**	**Population**
Allison 2012 [34] *(NR)*	105	University-based hospital in USA for women on community-based health insurance	African-American English speaking women, who were ≥ 18 years with a pre-gravid BMI of ≥25 kg/m^2^, had a singleton pregnancy; no pre-existing diabetes mellitus or autoimmune disorder, or regular use of steroid treatment
Brawarsky 2005 [9] *(NR)*	1100	Project WISH (Women and Infants Starting Healthy) cohort participants received prenatal care and planned to deliver at participating hospitals in San-Francisco, USA	Women who had a singleton, full-term birth (>37 weeks), identified their race as white, ‘black’, Latina, or Asian, and had complete pregnancy weight gain information, including a weight measured within four weeks of delivery
Chasan-Taber 2008 [36] *(2000–2003)*	770	Latina Gestational Diabetes Mellitus (GDM) cohort study based in public obstetrics/gynecology clinic and midwifery practice of a large tertiary care facility in Western Massachusetts, USA	Women who were Hispanic, 16–40 years old, <24 weeks, had a singleton pregnancy; no history of type 2 diabetes, hypertension or heart disease, chronic renal disease; treatment with medications thought to influence glucose tolerance adversely
Cogswell 1999 [37] (*1993*)	1661	Identified through a consumer mail panel (of 500, 000 households), representative of USA population in terms of geographic region, annual income, population density, household size and age	Women who had a singleton pregnancy, and were expecting to deliver within 3 months
Copper 1995 [39] *(1985–1988)*	1000	Data obtained from a prospective study of risk factors for fetal growth restriction that included pregnant women who delivered at the University of Alabama hospital, USA	Multiparous women, who had a live singleton birth at full term, predominantly ‘black’, and medically indigent; last available weight was within 2 weeks before delivery; oversampled women with one or more risk factors for Fetal Growth Restriction, including, but not limited to, smoking, a history of an low birth weight infant, and small stature
Herring 2008 [41] *(1999–2002)*	1537	Women recruited from project Viva having their first prenatal visit in one of eight urban and suburban obstetric clinics associated with multispecialty group practice in eastern Massachusetts, USA	Women with a fluency in English, <22 weeks, with a singleton; excluded: underweight women
Hill 2013 [42] (*NR)*	104	Participants recruited from Australian pregnancy online forum and in pregnancy and parenting magazines distributed at state and national level	Pregnant women >18 years and between 10 and 16 weeks
Laraia 2013 [43] *(2001–2005)*	1041 for univariate, 922 for multivariate	Pregnancy, Infection and Nutrition (PIN3) prospective cohort study recruited through the University of North Carolina Hospital and private physician obstetrics clinics in USA	Women ≥16 years, English speaking, planning to continue care or deliver at the study site and having a singleton pregnancy
Loris 1985 [44] *(1979–1982)*	46	Teen obstetric clinic of the University of Carolina Davis Medical Centre, USA	Teenagers delivering a singleton
McAnarney 1992 [45] *(1986–1989)*	116	Participants recruited from Rochester Study of Adolescent Pregnancy in New York, USA	Participants of a cohort of poor, ‘black’, 12 to 19 years
Mehta 2011 [46] *(2001–2005)*	1192	Pregnancy, Infection and Nutrition (PIN) cohort study delivering at the University of North Carolina Hospital, USA	Participants of a cohort who were >16 years, spoke English, were ≤20 weeks on their second prenatal visit, were planning to continue care or deliver at the study site, had access to a phone for telephone interviews, and were having singleton pregnancies
Mehta-Lee 2013 [63] *(2008–2010)*	775	Secondary analysis of data collected for two randomized-controlled trials of routine provider, primary care based breastfeeding promotion interventions in the Bronx, New York United States	English or Spanish speaking women >18 years, 1^st^ or 2^nd^ trimester of a singleton pregnancy without known risk factors for premature birth, medical contraindications to breastfeed, or infant conditions that prevent breastfeeding. Inclusion criteria for this study: medical record data for height, self-reported pre-pregnancy weight or a pregnancy weight <22 weeks, and a weight > 12 weeks later; exclusion: underweight
McPhie 2015 [62] *(NR)*	183	Participants were recruited via advertising on online mother, child and baby forums, in parenting magazines, at baby and children’s markets, and at obstetrician clinic waiting rooms in Geelong/Melbourne in the state of Vicotria, Australia	Women over the age of 18 years
Morling 2003 [47] *(NR)*	56 American women; 94 Japanese women	American women recruited from four obstetric clinics in the city of Schenectady, New York; Japanese women were recruited from the Centre of Obstetrics at the Central Hospital of Ethime	Women who were middle class, recruited during second trimester
Mumford 2008 [48] *(2001 to 2005)*	1223	PIN cohort study participant recruited from both public and private prenatal clinics at the University of North Carolina Hospital, USA	Participants who were ≥16 years, spoke English, ≤20 weeks on their second prenatal visit, planning to continue care or deliver at the study site and had a singleton pregnancy
Olson 2003 [49] *(NR)*	622	Women registered for prenatal care in a hospital and primary care clinic system serving a 10 county area of Upstate New York, USA	Women who entered prenatal care < third trimester, were ≥ 18 years at the time of delivery, planned to deliver within the local hospital and keep the baby, were healthy and mentally competent and gave birth to live singleton infants
Pomerleau^$^2000 [50] *(NR)*	68	Participants from studies at the Nicotine Research Laboratory, recruited from the general community in Michigan, USA	Women who had a first-born child age ≤10 years, singleton pregnancy, delivered at ≥ 37 weeks, smoked at least five cigarettes/day prior to first pregnancy, were smokers at the time of their first pregnancy (regardless of whether they quit during pregnancy or of their current smoking status); participants with a wide range of weight concerns and oversampled those who scored high on measures of dieting and bingeing severity
Steven-Simon 1993 [53] *(1986–1989)*	99	Participants were enrolled in the Colorado Adolescent maternity program at the University Hospital, USA	Participants who were 13 to 18 year old from diverse ethnic backgrounds
Steven-Simon 1995 [52] *(1986–1989)*	122	Participants of Rochester study of Adolescent pregnancy in New York, USA	Participants who were poor, ‘black’ and 12 to 19 years
Strychar^$^ 2000 [54] *(NR)*	115	Prenatal clinics at 3 university teaching hospitals in Montreal, Canada	Primiparous women with singleton pregnancies, ≥ 19 years; excluded: high-risk pregnancies, gestational diabetes, edema, preeclampsia, ‘black’ women and women of Asiatic and Hispanic origin
Sui 2013 [64] *(2010–2012)*	442	Prospective cohort study nested within the LIMIT (LIMITing weight gain in overweight an dobese women during pregnancy to improve health outcomes) randomized trial, evaluating the effect of an antenatal dietary and lifestyle intervention for women who are overweight or obese; public maternity hospitals in South Australian metropolitan area	Women with BMI >25 kg/m2 were recruited with a live singleton pregnancy form 10–20 weeks’ gestation at the time of their 1^st^ antenatal appointment
Tovar 2012 [55] *(2006–2011)*	952	Proyecto Buena Salud Cohort based in the public obstetrics and gynecology clinic and midwifery practice at a large tertiary care facility in Western Massachusetts, USA	Hispanic 16 to 40 year old women of Puerto Rican or Dominican Republic heritage (Caribbean Islanders), who were either themselves, or one of their a parent, or at least 2 of their grandparents born in the Caribbean Islands; women who had a full-term, live singleton birth; excluded: women with current use of medications that could influence glucose tolerance, history of diagnosis of diabetes prior to pregnancy, hypertension, heart disease or chronic renal disease
van der Wijden 2014 [66] *(2005–2006)*	161	Pregnant women from eight midwifery practices in The Netherlands were invited to participate in a randomized control trial on the New Life(style) intervention program consisting of five individual counselling session by one of two trained counsellors with the aim of preventing excess GWG. (Secondary analysis)	Women were eligible if <14 weeks pregnant (1^st^ ongoing pregnancy) and fluent in Dutch. Exclusion criteria for current study: pre-pregnancy BMI or objectively measured pregnancy weight gain could not be established, or if >/=2 items per scale missing in the Dutch Eating Behaviour Questionnaire or >/=3 items on other scales.
Walker^$^2002 [57] *(NR)*	305	Austin New Mothers Study cohort who completed the post-delivery panel in USA	White, African-American, and Hispanic low income women who could read and speak in English, were ≥ 18 years, had full term delivery (between 37 and 42 weeks based on medical records), singleton birth, no medical risks such as diabetes or hypertension during pregnancy, parity of ≤3, and who had Medicaid coverage for prenatal care
Webb 2009 [2] *(2001–2005)*	1605	PIN cohort study conducted in central North Carolina, USA	Women who were >16 years, had a singleton pregnancy, were <20 weeks at their second prenatal visit, had a live birth, and had GWG data
Wells 2006 [58] *(2000–2002)*	4528	Data from the Centre for Disease Control and Prevention’s Pregnancy Risk Assessment Monitoring System (PRAMS) for Colorado, USA	Women with live births, were ≥ 15 years
Wright^$^ 2013 [59] *(NR)*	101	Participants from Pennsylvania, USA. Details about the study setting not reported	Low income, English or Spanish speaking women who delivered a single live infant
Zhu 2013 [65] *(2008)*	1800	Women at Hefei Maternal and Child Health Hospital, Hefei, China	Women >32 weeks (retrospectively assessed stress in 1^st^ and 2^nd^ trimesters), singleton gestations. Exclusion criteria: >35 years, medically indicated preterm birth, birth defects, stillbirth, assisted reproductive technology, mental disorders, complications of pregnancy including diabetes, hypertension, heart failure, thyroid disease, intrahepatic cholestasis of pregnancy, moderate or severe anemia, history of abnormal pregnancy outcome including premature birth, spontaneous abortion, fetal death, stillbirth, birth defect, neonatal death
Zuckerman 1989 [60] *(1984–1987)*	1014	Prenatal clinic at Boston City Hospital, USA	Women who had the ability to communicate in English or Spanish, who gave informed consent

Note. ^$^Cross-sectional study; however, treated similar to a cohort study by authors; NR: Not Reported; GWG: Gestational Weight Gain.

**Table 3:** **Characteristics of included case–control and cross-sectional studies in systematic review of psychological antecedents of excess gestational weight gain**

**Author, year** ***(Years study span)***	**Sample size**	**Setting**	**Population**
Bagheri 2013 [35] *(2010)*	362	Women referred for prenatal care to a large women’s hospital in the south of Tehran, Iran	Fifteen to forty-six year-old pregnant women who were referred for prenatal care in a women’s hospital; >34 weeks and had a singleton pregnancy; cases were defined as pregnant women who gained weight in excess of Institute of Medicine guidelines and controls as women who gained weight within the guidelines; excluded: pregnant women with abnormal fetuses and those who received hormonal treatment during pregnancy or had diabetes, hypertension, thyroid or, renal chronic diseases
Conway^$^ 1999 [38] *(1995–1996)*	62	A large London hospital in United Kingdom	Caucasian women, who were expecting their first or second singleton baby, >18 years and free from known medical conditions which might affect nutrition or fetal outcomes
Dipietro^$^ 2003 [40] *(NR)*	130	Obstetric clinic in Baltimore, USA	Women with low risk, normal, singleton pregnancies, delivered at term, and with no history of smoking; predominantly well-educated, middle class women
McDonald^$^ 2013 [61] *(2012)*	330	Seven obstetrical and two midwifery clinics in southwestern Ontario, Canada	Women who had had at least one prenatal visit, could read English sufficiently well to complete the survey, and had a live singleton pregnancy
Sangi-haghpeykar^$^ 2013 [51] *(2011)*	282	Women delivering at a general hospital in Houston, USA	Women who were Hispanic, recruited immediately post-partum before leaving the hospital
Walker 2009 [56] *(2000–2003)*	1988	Pregnancy Risk Assessment Monitoring System (PRAMS) study data in New Mexico, USA	Hispanic mothers, ≥ 18 years, who had a singleton live birth during their most recent pregnancy, and had a full term (≤37 weeks) delivery

^$^Cross-sectional study; however, treated similar to a case–control study by authors; NR: Not Reported; GWG: Gestational Weight Gain.

### Quality assessment

#### Cohort studies

A detailed breakdown of the quality evaluation of the 29 included studies [2,9,34,36,37,39,41-50,52-55,57-60,62-66] is provided in supplementary information Additional file 1: Table S2. Four studies [44,50,54,58] had a score less than five. Women were generally representative of an average pregnant population in the community. In nine studies [37,41,44,47,54,58,59,63,65], fewer than half of the scales for the ascertainment of exposure were validated. The non-exposed cohort was almost always drawn from the same population. Follow-up was generally adequate in duration. Twenty-one studies [2,34-44,47,48,50,51,53-58,62,64-66] had a loss of 10% or greater at follow-up (or an unclear proportion), although the follow-up ranged from 48% to 95%. Eight studies were assessed as underpowered [36,42,50,52-54,57,59], and in two, the power could not be determined [2,62].

#### Case–control and cross-sectional studies

Detailed breakdown of quality evaluation of the six included studies [35,38,40,51,56,61] according to the modified Newcastle-Ottawa Scale for case–control studies is provided in supplementary information Additional file 1: Table S3. Two studies [38,40] had a score less than five. Only one study did not adjust for important confounders [38]. Five studies did not provide sufficient details about the response rate [35,40,51,56,61]. Two studies were underpowered [38,40].

There was insufficient information available in the included studies to construct funnel plots to assess publication bias.

#### Psychological factors and excess GWG

**Figure 2:**
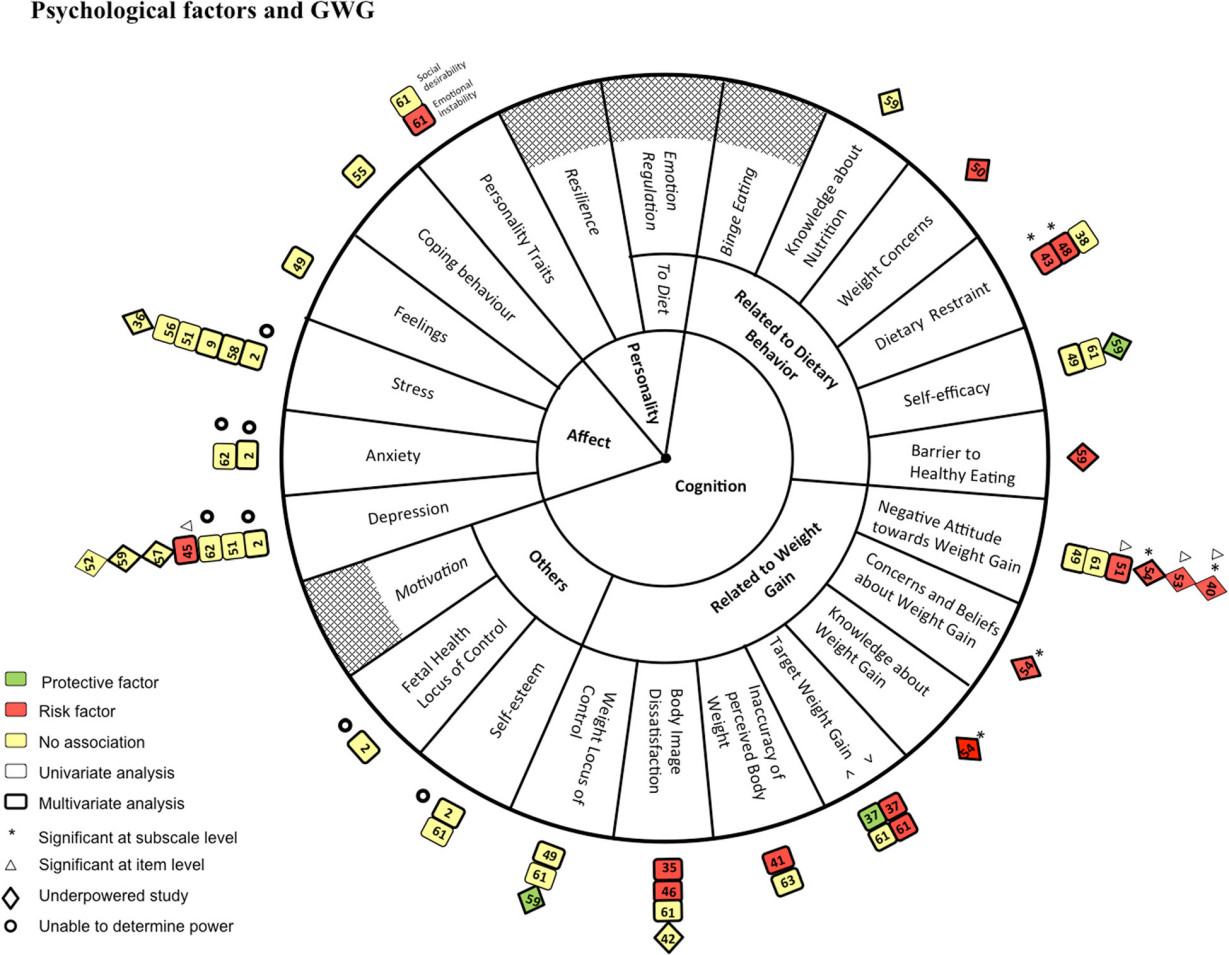
Diagrammatic representation (pinwheel) of relation between affect, cognition and personality and excess gestational weight gain in systematic review of psychological antecedents of excess gestational weight gain. The results of the systematic review on the relation of affect, cognitions and personality with excess GWG are depicted in Figure 2. The major domains are positioned in the innermost circle. In the next circle, cognition is further divided into three sub-domains. These sub-domains are further divided to represent the individual exposures investigated. Boxes () have been used to represent adequately powered studies and diamonds () represent underpowered studies. The number within the boxes and diamonds corresponds to each study’s reference as listed in Tables 4, 5, 6 and 7. Boxes and diamonds are further coded as follows: Studies which performed multivariate analysis are shown with a thick border; studies which performed univariate analysis are shown with a thin border; Green = significant negative association (protective factor), red = significant positive association (risk factor) and yellow = non-significant association. Adjacent to the boxes and diamonds, symbols represent the following:  = studies for which we were unable to determine power. * = significant only at subscale level of an exposure scale and △ = significant only at item level of an exposure scale. Exposures which were not investigated by any of the studies included in this systematic review are shown using crosshatching (). >: Greater than recommended target weight gain; <: Less than recommended target weight gain.

Results are shown for the three main psychological constructs (affect, cognitions related to weight gain, and cognitions related to dietary behavior) pertaining to our primary outcome, excess GWG (Figure 2, Tables 4, 5, 6 and 7, and Additional file 1: Figures S1,S2 and S3 and summarized in the section below.

**Table 4:** **Summary table of the relationship of affect and excess gestational weight gain during pregnancy in systematic review of psychological antecedents of excess gestational weight gain**

Author, Year (Study reference number)*	Scale used**, Validation	**Outcome**	**Crude (unadjusted) results**	**Adjusted results**	**Confounders adjusted for**	**Summary of results**
**Exposure: Depression**						
McAnarney 1992 [45]	Centre for Epidemiological Studies-Depression Scale (CES-D), validated	Rate of weight gain categorized as slow, average and rapid	Mean (SD) CES-D in each weight gain category: 22 (±9); 20 (±7); 24 (±8) (p <0.05)	OR (95% CI ) of rapid weight gain:	Covariates used but not reported	Only 1 item was significant on multivariate analysis ➔
			Item: Suicidal thoughts and attempts	Item: ‘Suicidal thoughts and attempts’ 5.0 (1.28 to 19.57)		
			Proportion within each weight gain category 13%; 4.6%; 19.4% (p <0.05)			
McPhie 2015 [62]	Depression, Anxiety, and Stress Scale-21 (DASS-21), validated	Excess GWG	In 1^st^ trimester, mild depression in 8.5% of those who gained in excess and 8.9% who gained within guidelines (for moderate depression, 2.8% and 0.9%, respectively); mild anxiety in 9.9% and 11.6%, respectively (for moderate anxiety, 7.0% and 0.9%, respectively)	NA	NA	NS on univariate analysis
Sangi-Haghpeykar 2013 [51]	Patient Health Questionnaire (PHQ), validated	Excess GWG	Proportion with GWG categories: 9%, 9% (p-value NS)	NA	NA	NS on univariate analysis; variable not entered in the multivariate study
Steven-Simon 1995 [52]	CES-D, validated		Effect estimate not reported; (p-value NS)	NA	NA	NS on univariate analysis
						Multivariate analysis was not done
Walker 2002 [57]	CES-D, validated	Excess GWG	Correlation co-efficient (p-value): r = 0.02 (p-value NS)	β (SE): 0.0 (0.1)	Pre-pregnancy BMI, age, parity, ethnicity, newborn gender, maternal height, food habits	NS on univariate or multivariate analyses
Webb 2009 [2]	CES-D, validated	Excess GWG; Adequacy Ratio	RR (95% CI ):	RR (95% CI ):	Pre-gravid BMI, other socio-demographic, dietary and physical activity covariates	NS on univariate or multivariate analyses; Adequacy ratio outcome was significant only on univariate analysis
			**CES-D score (<20 weeks)**	**CES-D score (<20 weeks)**		
			Low 1.0 (Reference); Moderate 1.06 (1.0 to 1.2); High 1.03 (0.9 to 1.1)	Low 1.0 (Reference); Moderate 1.01 (0.9 to 1.1); High 0.98 (0.9 to 1.1) (p = 0.91)		
			**CES-D score (24–29 weeks)**	**CES-D score (24–29 weeks)**		
			Low 1.0 (Reference); Moderate 1.08 (1.0 to 1.2); High 1.12 (1.0 to 1.1)	Low 1.0 (Reference); Moderate 1.02 (0.9 to 1.1); High 1.02 (0.9 to 1.1) (p = 0.76)		
Wright 2013 [59]	Edinburgh Postnatal Depression Scale (EPDS), validated	Excess GWG ^$;^ GWG (continuous)^$$^;	β (95% CI ): 0.88 (0.1 to 1.7)	Effect estimate not reported for excess GWG	Pre-pregnancy BMI, age, race	Results were reported to be similar to secondary outcome but estimates were not reported, hence considered non-significant on univariate or multivariate analysis
				β (95% CI) for secondary outcome: 0.3 (−1.0 to 1.5)		
**Exposure: Anxiety**						
McPhie 2015 [62]	Depression, Anxiety, and Stress Scale-21 (DASS-21), validated	Excess GWG	In 1^st^ trimester, mild anxiety in 9.9% of those who gained in excess and 11.6% who gained within guidelines, respectively (for moderate anxiety, 7.0% and 0.9%, respectively)	NA	NA	NS on univariate analysis
Webb 2009 [2]	State and Trait Anxiety Inventory (STAI), validated	Excess GWG; Adequacy Ratio	RR (95% CI):	RR (95% CI):	Pre-gravid BMI, other socio-demographic, dietary and physical activity covariates	NS on univariate or multivariate analyses; adequacy ratio was also NS on univariate or multivariate analyses
			**STAI-T (<20 weeks)**	**STAI-T (<20 weeks)**		
			Low 1.0 (Reference); Moderate 1.04 (1.0 to 1.1); High 0.98 (0.9 to 1.1)	Low 1.0 (Reference); Moderate 1.02 (1.0 to 1.1); High 1.01 (1.0 to 1.1)		
			**STAI-S (<20 weeks)**	**STAI-S (<20 weeks)**		
			Low 1.0 (Reference); Moderate 0.94 (0.9 to 1.0); High 0.94 (0.9 to 1.0)	Low 1.0 (Reference); Moderate 1.06 (1.0 to 1.1); High 1.00 (0.9 to 1.1)		
			**STAI-S (24–29 weeks)**	**STAI-S (24 to29 weeks)**		
			Low 1.0 (Reference); Moderate 1.00 (0.9 to 1.1); High 0.95 (0.9 to 1.0)	Low 1.0 (Reference); Moderate 1.01 (0.9 to 1.1); High 0.99 (0.9 to 1.1)		
**Exposure: Stress**						
Brawarsky 2005 [9]	Perceived Stress Scale-PSS (short form), validated	Excess GWG	Proportion within GWG categories:	NA	NA	NS on univariate or multivariate analyses
			Stress categorised as:			
			Yes: 46.4%, 32.2%			
			No: 55.4%, 32.2%			
Chasan-Taber 2008 [36]	Perceived Stress Scale-PSS (short form), validated	Excess GWG		OR (95% CI ):	Pre-pregnancy BMI, parity, age, generation in USA, prenatal care, caloric intake, household activity	NS on univariate or multivariate analyses
			Proportion within GWG categories:	Maternal stress categorised as:		
			0-2: 51.5%, 25.0%;	0-2: 1.0 (Reference);		
			3-5: 39.5%, 38.4%;	3-5: 0.5 (0.3 to 0.9);		
			6-8: 43.4%, 34.4%;	6-8: 0.6 (0.4 to 1.1);		
			≥9: 51.3%, 28.6%;	≥9: 0.9 (0.5 to 1.6);		
			(p for trend = .75 and .82, respectively)	Missing: 1.1 (0.4 to 3.2)		
Chasan-Taber 2008 [36]	PRAMS standard questions – based on modified Life Event Inventory, validated	Excess GWG	Proportions within GWG categories:	NA	NA	NS on univariate analysis; variable not entered in a multivariate model
			Number of life events categorised as:			
			None: 46.4%, 33.6%;			
			1: 46.0%, 35.4%			
			2: 50.0%, 28.8;			
			≥3: 42.6%, 31.1%			
			(p for trend = .51 and .37 respectively)			
Sangi-haghpeykar 2013 [51]	Prenatal Psychosocial Profile Hassles Scale, validated	Excess GWG ^$ and $$^	Mean (±SD):	NA	NA	NS on univariate analysis; Variable not entered in the multivariate model
			13.7 (±2.8), 14.4 (±4.0)			
Walker 2009 [56]	PRAMS standard questions – based on modified Life Event Inventory, validated (18 items were used)	Excess GWG	Proportions within GWG categories:	NA	NA	NS on univariate analysis; Variable not entered in the multivariate model
			Maternal stress categorised as:			
			None: 20.93%, 18.48%			
			1-2: 38.76%, 40.65%;			
			3-5: 32.11%, 31.49%;			
			6-18: 8.20%, 9.39%			
Webb 2009 [2]	Perceived Stress Scale (PSS), validated	Excess GWG	RR (95% CI ):	RR (95% CI ):	Pre-gravid BMI, other socio-demographic, dietary and physical activity covariates	NS on univariate or multivariate analyses; NS results for adequacy ratio outcome
			**PSS 17–22 weeks**	**PSS 17–22 weeks**		
			Low 1.0 (Reference);	Low 1.0 (Reference);		
			Moderate 0.99 (0.9 to 1.0);	Moderate 0.99 (0.9 to 1.0);		
			High 1.03 (1.0 to 1.1) ;	High 0.99 (0.9 to 1.1)		
			**PSS 27–30 weeks**	**PSS 27–30 weeks**		
			Low 1.0 (Reference);	Low 1.0 (Reference);		
			Moderate 1.04 (1.0 to 1.1);	Moderate 1.01 (1.0 to 1.1);		
			High 1.07 (1.0 to 1.2)	High 1.01 (1.0 to 1.1)		
Wells 2006 [58]	PRAMS standard question – based on modified Life Event Inventory, validated (13 items were used)	Excess GWG	Proportions within GWG categories:	OR (95% CI ):		NS on univariate level or multivariate analyses
			Maternal stress categorised as:	0 Stressor: 1.0 (Reference);		
			0 Stressors: 41.3%, 36.4%;	1-2 stressors: 1.03		
			1-2 Stressors: 41.7%, 36.2%;	(0.84 to 1.26);		
			3 or more stressors: 39.9%, 32.5%	≥3 stressors: 1.04 (0.82 to 1.32)		
**Exposure: Feelings**						
Olson 2003 [49]	Investigator developed series of statement on Feelings about motherhood, Not validated	Excess GWG ^$$^	Proportion of exposure within Excess GWG category:	NA	NA	NS on univariate analysis; variable not entered in a multivariate model
			Low 43.8%;			
			Medium 37.1%			
			High 41.9%			
**Exposure: Coping behavior**						
Tovar 2012 [55]	Psychological Acculturation Scale, validated	Excess GWG ^$^	Proportions within GWG categories:	OR (95% CI ):	Pre-pregnancy weight, age, parity, perceived stress, gestational age and physical activity	NS on univariate or multivariate analysis; NS association with other weight gain outcomes (rate of weight gain, weight gain as continuous)
			Low acculturation	Continuous acculturation score		
			49.3%, 30.6%;	1.0 (0.8 to 1.3)		
			Medium acculturation			
			42.2%, 31.1%;			
			High acculturation			
			47%, 31.5%			
			(p = 0.4)			

*Study reference number correspond to those cited in a pinwheel and web plot; **Scale details can be found in Additional file 1: Table S1; ^$^2009 IOM GWG guidelines; ^$$^ GWG measured in pounds (lb); ➔Positive association (Risk factor);  Negative association (Protective factor); BMI: Body Mass Index; CES-D: Centre for Epidemiological Studies-Depression Scale; GWG: Gestational Weight Gain; NA: Not Applicable; NR: Not Reported; NS: Not Significant; PHQ: Patient Health Questionnaire; PRAMS: Pregnancy Risk Assessment Monitoring System; PSS: Perceived Stress Scale; STAI-S: State and Trait Anxiety Inventory-State; STAI-T: State and Trait Anxiety Inventory-Trait.

**Table 5:** **Summary table of the relationship between cognitions related to weight gain and excess gestational weight gain in systematic review of psychological antecedents of excess gestational weight gain**

Author, year (Study reference number)*	Scale used**, Validation	**Outcomes**	**Crude(unadjusted) results**	**Adjusted results**	**Confounders adjusted for**	**Summary of results**
**Exposure: Negative attitude towards weight gain**						
DiPietro 2003 [40]	Pregnancy and Weight Gain Attitude Scale, validated	Excess GWG	Proportions within GWG categories (p-value):	NA	Pre-pregnancy BMI	Only 1 item and two sub-scales were significant on univariate analyses ➔
			**Individual items:**			
			-Embarrassed about weight			
			28%, 8% ( p <0.05)			
			-Worried will get fat			
			43%, 37% (p-value NS)			
			Feel unattractive			
			28%, 14% (p-value NS)			
			-Embarrassed when nurse weight me			
			21%, 21% (p-value NS)			
			-Cannot wear what is in style			
			18%, 27% (p-value NS)			
			**Subscales:**			
			Negative pregnancy body image r = 0.28 (p < 0.001)			
			Pregnancy experience scale r = 0.20 (p < 0.001)			
McDonald 2013 [61]	Pregnancy and Weight Gain Attitude Scale, validated (Attitude towards weight gain scale)	Excess GWG	Mean (SD) in those gaining above 17.4 (3.4) vs within 17.9 (2.8); OR 0.95 (0.86 to 1.05)	NA	NA	NS on univariate therefore not included in multivariate
Olson 2003 [49]	Pregnancy and Weight Gain Attitude Scale, validated	Excess GWG ^$$^	Effect estimate not reported; (p-value NS)	NA	NA	NS on univariate analysis
		(^$$$^modified 1990 Institute of Medicine guidelines)				Variable not entered in the multivariate model
Sangi-haghpeykar 2013 [51]	Pregnancy and Weight Gain Attitude Scale, validated	Excess GWG ^$^	Proportions within GWG categories (p-value):	OR (95% CI )	Pre-pregnancy BMI, USA born, unmarried	Only a few items were significant on univariate or multivariate analyses ➔
			**Individual items**			
			-Worried will get fat: 28%, 15% ( p <0.05)	-Embarrassed when nurse weighed me: 4.61 (1.18 to 29.80)		
			-Embarrassed when nurse weighed me: 14%, 3% ( p <0.05)	-Don’t care how much I gain: 3.80 (1.47 to 11.36)		
			-Don’t care how much I gain: 23%, 9% (p <0.05)			
Stevens-Simon 1993 [53]	Pregnancy and Weight Gain Attitude Scale, validated	Rate of weight gain categorised into slow (<0.23 kg/wk), average (0.23 – 0.4 kg/wk), rapid (>0.4 kg/wk)	Correlation co-efficient (p-value):	NA	NA	Only a few items were significant on univariate analyses ➔
			Total scale score			Multivariate analysis was not done
			r = 0.12 (p <0.14)			
			**Mean (± SD) attitude score among three outcome categories**			
			3.4(±0.6), 3.5(±0.5), 3.5(±0.6) (p >0.05)			
			**Individual items** (Correlation co-efficient not reported):			
			-Liked wearing maternity clothes: (p <0.05)			
			-Felt unattractive: (p <0.05)			
			-Embarrassed when nurse weighed me: (p <0.05)			
			-Cannot wear what is in style: (p <0.05)			
Strychar 2000 [54]	Investigator developed, Not validated	Excess GWG	NR	**Sub-scale** – less favourable attitude towards weight gain led to excess weight gain	Pre-pregnancy BMI, age, marital status, education, smoking, and alcohol	Only a sub-scale was significant on multivariate analysis ➔
				Effect estimate not reported (p <0.05		
**Exposure: Concerns and beliefs about weight gain**						
Strychar 2000 [54]	Investigator developed, Not validated	Excess GWG	NR	**Sub-scale:** Perceived concern about their weight – more concerned leads to excess weight gain Effect estimate not reported; (p <0.05)	Pre-pregnancy BMI, age, marital status, education, smoking, and alcohol	Only a sub-scale, namely, ‘perceived concern’ was significant on multivariate analysis ➔
**Exposure: Knowledge about weight gain**						
Strychar 2000 [54]	Investigator developed, Not validated	Excess GWG	NR	**Sub-scale:** Importance of not gaining an excess amount of weight– Less knowledge leads to excess weight gain	Pre-pregnancy BMI, age, marital status, education, smoking, and alcohol	Only a sub-scale, namely, ‘ importance of not gaining an excess amount of weight’ was significant on multivariate analysis ➔
				Effect estimate not reported; (p <0.05)		
**Exposure: Target weight gain**						
Cogswell, 1999 [37]	Investigator developed single item; Not validated	Excess GWG	NR	OR (95% CI )	Pre-pregnancy BMI, maternal height, age, race, education, marital status, parity, prenatal care, WIC participants,, income	Significant on multivariate analysis
				Target weight gain categories		➔ (> recommended)
				<Recommended 0.4 (0.2 to 0.6)		(< recommended)
				Recommended 1.0 (Reference)		
				>Recommended 6.1 (4.1 to 8.9)		
McDonald 2013 [61]	Investigator developed single item; Investigator developed, not validated	Excess GWG	OR (95% CI )	OR (95% CI )	Pre-pregnancy BMI group, first birth, planned	Planned gain above the guidelines Significant on both univariate and multivariate analysis
			Planned gain above the guidelines 9.31 (3.86 to 22.42), planned gain below 0.78 (0.33 to 1.84)	Planned gain above the guidelines 11.18 (4.45 to 28.06); planned gain below 0.69 (0.26 to 1.80)	weight gain, daily soda or juice consumption, watching television before bedtime, locus of control to Eysenck’s neurotic scale of emotional instability, and satisfaction with pre-pregnancy weight	➔ (> recommended) planned gain below NS on univariate or multivariate multivariate
**Exposure: Inaccuracy of perceived body weight**						
Herring 2008 [41]	Previously published single item adopted National Health and Nutrition Examination Survey, No reference to validation	Excess GWG	Proportion of Excess GWG within each exposure category:	OR (95% CI ):	Pre-pregnancy BMI, age, education, marital status, income, employment, ethnicity, parity, smoking, gestational length	Significant on univariate or multivariate analyses ➔
			Normal weight, accurate assessor 47%	Normal weight, accurate assessor 1.0 (reference);		
			Normal weight, over-assessor 57%	Normal weight, over-assessor 2.0 (1.3 to 3.0);		
			Overweight, accurate assessor 62%	Overweight accurate assessor 2.9 (2.2 to 3.9);		
			Overweight under-assessor 81% (p <0.05)	Overweight under-assessor 7.6 (3.4 to 17.0)		
Mehta-Lee 2013 [63]	Single item, Perceived weight status was defined as “accurate” or “inaccurate” based upon the level of concordance between BMI (derived from actual weight) and self reported overweight or obesity (no reference to validation)	Excess GWG	OR (95% CI ): Inaccurate reporters 1.2 (0.8, 1.8);	OR (95% CI ): Inaccurate reporters 1.1 (0.7, 1.7);	Stratified by BMI; adjusted for: WIC status, employment status, race, native born, smoking, parity and either pre-gestational or gestational diabetes	NS on univariate and on multivariate analyses
**Exposure: Body image dissatisfaction**						
Bagheri 2013 [35]	Body Image Assessment for Obesity (BIA-O), Validated	Excess (cases) vs. Adequate (controls) GWG	OR (95% CI ):	OR (95% CI ):	Pre-pregnancy BMI, age, parity, social class, energy intake	Significant on univariate or multivariate analyses ➔
			Heavier body size preference 0.54 (0.27 to 1.04)	Heavier body size preference 0.44 (0.18 to 1.10)		
			Thinner Body Size Preference 2.17 (1.17 – 4.02)	Thinner body size preference 3.12 (1.97 to 4.95)		
Hill 2013 [42]	Body Attitude Questionnaire (BAQ), Validated, modified	Excess GWG ^$^	NR	Effect estimates were not reported; p-value NS	Pre-pregnancy BMI, age, parity, education level	NS on multivariate analysis
Mehta 2011 [46]	Body Image Assessment for Obesity (BIA-O), Validated	Excess GWG	RR (95% CI ):	RR (95% CI ):	Pre-pregnancy BMI	Significant on multivariate analysis ➔
			Heavier body size preference 1.79 (0.52-9.58)	Thinner body size preference		
			Thinner body size preference 0.88 (0.82 to 0.94)	<16 years of education 1.11 (1.00 to 1.22)		
				≥16 years of education 0.92 (0.83 to 1.01)		
McDonald 2013 [61]	Satisfaction with pre-pregnancy weight , not stated if validated or not	Excess GWG	OR (95% CI ):	NA	NA	Significant on univariate analysis
			Not or not at all satisfied vs. satisfied or very satisfied 0.25 (0.10 to 0.60)			NS on multivariate analysis
**Exposure: Weight Locus of Control**						
McDonald 2013 [61]	Locus of control score, validated	Excess GWG	OR (95% CI ) 1.12 (1 to 1.26)	NA	NA	NS on univariate analysis; Variable not entered in the multivariate model
Olson 2003 [49]	Weight Locus of Control (WLOC), Validated	Excess GWG ^$$^	Effect estimate not reported; p-value NS	NA	NA	NS on univariate analysis; Variable not entered in the multivariate model
Wright 2013 [59]	Single item from Attitude towards weigh gain scale by Palmer, Validated, modified	Excess GWG;	Effect estimate not reported for Adequacy ratio	Effect estimate not reported for Excess GWG	Pre-pregnancy BMI, age, race	Results were reported to be similar to secondary outcome , hence considered significant on univariate or multivariate analysis
		GWG (continuous)^$$^	β (95% CI ) for secondary outcome:-11.6 (−21.4 to −1.9)	β (95% CI ) for secondary outcome: −16.1 (−28.7 to −3.4)		

*Study reference number correspond to those cited in a pinwheel and web plot; **Scale details can be found in Additional file 1: Table S1; ^$^2009 IOM GWG guidelines; ^$$^ GWG measured in pounds (lb); ➔ Positive association (Risk factor);  Negative association (Protective factor); ^$$$^ For obese women, upper limit of recommended weight gain was set as same as that of the overweight women; BAQ: Body Attitude Questionnaire; BIA-O: Body Image Assessment for Obesity; BMI: Body Mass Index; GWG: Gestational Weight Gain; NA: Not Applicable; NR: Not Reported; NS: Not Significant;

**Table 6:** **Summary table of the relationship of cognitions related to dietary behavior to excess gestational weight gain in systematic review of psychological antecedents of excess gestational weight gain**

Author, year (Study reference number)*	Scale used**, Validation	**Outcome(s)**	**Crude (unadjusted) results**	**Adjusted results**	**Confounders adjusted for**	**Summary of results**
**Exposure: Knowledge about nutrition**						
Wright 2013 [59]	Investigator developed, Validated	Excess GWG;	β (95% CI ):	Effect estimate not reported for excess GWG	Pre-Pregnancy BMI, age, race	Results were reported to be similar to secondary outcome but estimates were not reported. Hence considered NS on univariate or multivariate analyses
		GWG (continuous)^$$^	−1.2 (−3.2 to 0.69)	β (95% CI ) for secondary outcome: −0.14 (−2.8 to 2.5)		
**Exposure: Weight concerns**						
Pomerleau 2000 [50]	Dieting and Binge Eating Severity Scale (DBESS), Validated	Difference between actual and current maximum recommended weight gain (continuous)	Mean (± SD) excess GWG between two weight concern categories:	Effect estimates not reported	NR	Significant on multivariate analysis; weight gain (lb) as a continuous outcome also has a positive significant association with weight concern categories ➔
			Low Weight Concern ;=2.9 (±12.7);	ANOVA F-test statistics = 7.614 (p <0.01)		
			High Weight Concern 15.6 (±21.9) (p <0.01)			
**Cognitive dietary restraint**						
Conway 1999 [38]	Revised Restraint Scale (RRS), Validated	Excess GWG	Proportions with GWG categories (p-value):	NA	NA	NS on univariate analysis
			Dietary Restraint (Full scale) 48%, 30% (p = 0.07);			Multivariate analysis was not done
			Weight Fluctuation subscale 46%, 31% (p = 0.054);			
			Concern for dieting subscale 50%, 33% (p = 0.601)			
Laraia 2013 [43]	RRS, Validated	Excess GWG for univariate;	Proportion within GWG category:	β (95% CI ):	Pre-pregnancy BMI, maternal race, age, income, education, marital status, parity, gestational age, smoking, physical activity in 1^st^ trimester	Full scale was significant on univariate or multivariate analyses; subscales were significant on multivariate analysis ➔
		Adequacy Ratio for univariate and multivariate	Low dietary Restraint Food secure 52.7%, 35.4%;	Interaction between Marginally Food Insecure and:		
			Marginally food insecure 52.7%, 25.5%	High Restraint 0.53 (0.33 to 0.73)		
			High dietary Restraint Food secure 71.5%,	Dieters 0.50 (0.30 to 0.70)		
			16.8%;	Weight Cyclers 0.54 (0.34 to 0.74)		
			Marginally food insecure 74.0%, 11.0%			
			Overall х^2^(p-value ^)^ :57.3 (p <0.001)			
Mumford 2008 [48]	RRS, Validated	Adequacy Ratio	NR	OR (95% CI ):	Pre-pregnancy BMI, race, education, poverty, physical activity, weight gain attitude	Only subscales were significant on multivariate analyses ➔
				**Overall**		
				Restrained eating 1.12 (0.94 to 1.31)		
				Non-Restrained eating 0.95 (0.78 to 1.12)		
				**Dieters vs. Non-Dieters**		
				Underweight 0.94 (0.68 to 1.19); 1.02 (0.89 to 1.16);		
				Normal Weight 1.50 (1.40 to 1.60); 1.31 (1.23 to 1.40); Overweight 1.97 (1.80 to 2.15); 1.79 (1.54 to 2.03);		
				Obese 2.09 (1.98 to 2.21); 1.73 (1.53 to 1.93)		
				**Cyclers vs. Non-Cyclers**		
				Underweight 0.88 (0.66 to 1.11); 0.94 (0.77 to 1.11);		
				Normal Weight 1.38 (1.25 to 1.52);		
				1.25 (1.12 to 1.37); Overweight 1.92 (1.72 to 2.12); 1.58 (1.35 to 1.80);		
				Obese 2.11 (1.96 to 2.26); 1.73 (1.54 to 1.91)		
**Exposure: Self-efficacy**						
McDonald 2013 [61]	Self-efficacy in achieving healthy weight, ii) towards controlling food Intake; iii) towards weight Management, not stated if validated	Excess GWG	OR (95% CI ):	NA	NA	NS on univariate Analysis;
			0.97 (0.92 to 1.02); ii) 0.91 (0.79 to 1.05); iii) 0.94 (0.86 to 1.03)			not entered in the multivariate model
Olson 2003 [49]	Investigator Developed, Not validated	Excess GWG ^$$^	Effect estimate not reported (p-value NS)	NA	NA	NS on univariate analysis; variable not entered in the multivariate model
Wright 2013 [59]	Investigator developed, Not validated	Excess GWG; GWG (continuous)^$$^	Effect estimate not reported for excess GWG	Effect estimate not reported for excess GWG	Pre-pregnancy BMI, age, race	Results were reported to be similar to secondary outcome but Estimates were not reported, hence considered significant on univariate or multivariate analysis
			β (95% CI ) for secondary outcome:	β (95% CI ) for secondary outcome: −3.6 (−6.8 to −0.3)		
			β (95% CI ) -1.3 (−2.6 – 0.0)			
**Exposure: Barriers to healthy eating**						
Wright 2013 [59]	Fowles’ Barriers to Health Eating Scale (BHES), Validated	Adequacy ratio; Excess GWG ^$$^	β (95% CI ):	β (95% CI ):	Pre-pregnancy BMI, age, race	Results were reported to be similar to secondary outcome but estimates were not reported, hence considered significant on multivariate analysis ➔
			0.12 (−0.6 to 0.8)	2.0 (0.3 to 3.7)		

*Study reference number correspond to those cited in a pinwheel and web plot; **Scale details can be found in Additional file 1: Table S1; ^$^2009 IOM GWG guidelines; ^$$^ GWG measured in pounds (lb); ➔Positive association (Risk factor);  Negative association (Protective factor); ANOVA: Analysis of Variance; BMI: Body Mass Index; GWG: Gestational Weight Gain; NA: Not Applicable; NS: Not Significant; RRS: Revised Restraint Scale.

**Table 7:** **Summary table of the relationship between personality and ‘other’ cognitions, and excess gestational weight gain, in systematic review of psychological antecedents of excess gestational weight gain**

Author, year (Study reference number)*	Scale used**, Validation	**Outcome(s)**	**Crude (unadjusted) results**	**Adjusted results**	**Confounders adjusted for**	**Summary of results**
**Exposure: Personality Traits**						
McDonald 2013 [61]	Eysenck’s Neurotic Scale of Emotional Instability (Personality trait), validated;	Excess GWG	OR 95% CI 1.24 (1.11 to 1.39) (per unit increase on scale)	OR 95% CI 1.26 (1.10 to 1.44) (per unit increase on scale)	pre-pregnancy BMI group, first birth, planned weight gain, daily soda or juice consumption, watching television before bedtime, locus of control to Eysenck’s neurotic scale of emotional instability, and satisfaction with pre-pregnancy weight	Neurotic Scale of Emotional Instability Significant on univariate analysis and multivariate ➔
						Lie Scale of Social Desirability NS on univariate; not included in multivariate
McDonald 2013 [61]	Eysenck’s Lie Scale of Social Desirability (Personality trait), validated	Excess GWG	OR 95% CI 1.24	NA	NA	NS on univariate; Not entered into multivariate analyses
			0.95 (0.84 to 1.08)			
**Exposure: ‘Other’ Cognitions (Fetal Health Locus of Control)**						
Webb 2009 [2]	Fetal Health Locus of Control (FHLC), Validated	Excess GWG;	RR (95% CI ):	RR (95% CI ):	Pre-pregnancy BMI and other identified maternal socio-demographic, dietary and physical activity variables (exact variables not reported)	NS on univariate or multivariate analyses; similar results for adequacy ratio outcome
		Adequacy ratio	**FHLC-(Internality scale)**	**FHLC--(Internality Scale)**		
			Low 1.07 (1.0 to 1.2)	Low 1.02 (1.0 to 1.1)		
			Moderate 1.03	Moderate 1.01 (0.9		
			(0.9 to 1.1)	to 1.1)		
			High 1.0	High 1.0		
			(Reference)	(Reference)		
			**FHLC-(Powerful**	**FHLC-(Powerful**		
			**others scale)**	**others scale)**		
			Low 1.0 (Reference)	Low 1.0 (Reference)		
			Moderate 1.10 (1.0 to 1.2)	Moderate 1.00 (0.9 to 1.1)		
			High 1.05 (1.0 to 1.1)	High 0.96 (0.9 to 1.0)		
			**FHLC-(Chances scale)**	**FHLC-(Chances scale)**		
			Low 1.0 (Reference)	Low 1.0 (Reference)		
			Moderate 1.07 (1.0 to 1.2)	Moderate 1.00 (0.9 to 1.1)		
			High 1.08 (1.0 to 1.2)	High 1.01 (0.9 to 1.1)		
**Exposure: ‘Other’ Cognitions (Self-esteem)**						
McDonald 2013 [61]	Robins Self-esteem scale, validated	Excess GWG	OR (95% CI ) for “Not very true” vs. other in terms of positive self esteem 0.28 (0.04 to 2.19)	NA	NA	NS on univariate therefore not included in multivariate
Webb 2009 [2]	Self-esteem scale, Previously published, no reference to validation	Excess GWG;	RR (95% CI )	RR (95% CI )	Pre-pregnancy BMI, other socio-demographic, dietary and physical activity covariates	NS on univariate or multivariate analyses;
		Adequacy Ratio	Low 1.01 (0.9 to 1.1);	Low 0.99 (0.9 to 1.1);		NS results for adequacy ratio outcome
			Moderate 1.03 (1.0 to 1.1);	Moderate 1.02 (0.9 to 1.1);		
			High 1.0 (Reference)	High 1.0 (Reference)		

*Study reference number corresponds to those cited in a pinwheel and web plot; **Scale details can be found in Additional file 1: Table S1; FHLC: Fetal Health Locus of Control; NA: Not Applicable. ➔ Positive association (Risk factor).

#### Affect and excess GWG

Overall, affective states were not found to be related to excess GWG (Figure 2, Table 4, and Additional file 1: Figure S1). Only one of the six studies examining depression [45], found a significant association and this was between a severe measure of depression ‘suicidal thoughts and attempts’ and excess GWG (among ‘black’ adolescents; OR 5.0; 95% CI 1.28 to 19.57). Only one [65] of seven studies found a negative association between stress and excess GWG [2,9,36,51,56,58], however this study retrospectively assessed first trimester stress when the participants were at least 32 weeks gestation There was no association between excess GWG and feelings about motherhood [49], psychological acculturation (as a measure of coping behavior) [55] or anxiety [2].

#### Cognitions related to weight gain and excess GWG

Variable associations were found between cognitions related to weight gain and excess GWG. Of the eight cognitions related to weight gain, most were investigated by single studies while three were examined by more than one study (Figure 2, Table 5, and Additional file 1: Figure S2). Target weight gain ‘greater than recommended’ increased the risk of excess GWG (OR 6.1; 95% CI 4.1 to 8.9), whereas target weight gain ‘less than recommended’ decreased the risk (OR 0.4; 95% CI 0.2 to 0.6) of excess GWG [37], and similar associations were also found in a cross-sectional study [61]. Inaccuracy of perceived pre-pregnancy body weight was a risk for excess GWG among both normal weight women (OR 2.0; 95% CI 1.3 to 3.0) and overweight women (OR 7.6; 95% CI 3.4 to 17.0) [41] but was not in another study [63]. Less knowledge about the ‘importance of not gaining too much weight’ and more ‘perceived concern’ about weight gain were risk factors for excess GWG [54]. A non-significant association was found between self-esteem and excess GWG in two studies [2,61] .

Two of four studies found an association between body image dissatisfaction (preference for thinner body size) and excess GWG; one [35] noted this in their whole population (OR 3.12; 95% CI 1.89 to 4.95), whereas it held true only in women with less than 16 years of education in the other (RR 1.11; 95% CI 1.00 to 1.22) [46]. Two other studies reported non-significant associations [42,61], potentially due to lack of statistical power, given that the examination of GWG as a continuous outcome was significantly associated with the attractiveness subscale in one of the studies [42].

Four of six studies reported that a negative attitude towards weight gain, at a subscale or individual item level, was associated with excess GWG [40,51,54] or a rapid rate of weight gain [53] although two of these studies examined the association only with univariate analysis [40,53], while two others reported a non-significant effect [49,61] .

Weight locus of control appeared as a protective factor in one study [59], but had a non-significant effect in the other two studies [49,61] .

#### Cognitions related to dietary behavior and excess GWG

Variable associations were found between cognitions related to dietary behavior and excess GWG. Positive associations were found in two studies examining weight concerns [50] and barriers to healthy eating [59] with excess GWG, but both studies were underpowered (Figure 2, Table 6, and Additional file 1: Figure S3). Knowledge about nutrition was not significantly associated with excess GWG [59]. Two studies found no association between self-efficacy and excess GWG [49,61], and a third reported a negative association [59].

One of three studies found a positive association between dietary restraint on the full scale as well as on the two sub-scales of dietary restraint, namely dieters and weight cyclers, with excess GWG [38,43,48]. Laraia [43] found a positive association between high dietary restraint (OR 1.65; 95% CI 1.35 to 2.01), dieting (OR 1.72; 95% CI 1.40 to 2.10) and weight cycling (OR 1.70; 95% CI 1.39 to 2.08) and excess GWG for marginally food insecure women only. A second study [48] found a significant association between the dieting and weight cycling sub-scales (although not the full scale) and the adequacy of weight gain ratio. A third study [38] found no association between dietary restraint and excess GWG but only performed univariate analyses.

#### Personality and excess GWG

The only study to examine personality found a positive association between higher emotional instability and excess GWG, but no association with the social desirability scale [61] (Figure 2 and Table 7).

#### ‘Other’ psychological factors in cognitions and excess GWG

One study [2] found non-significant associations between three sub-scales of fetal health locus of control (i.e., internality, powerful others and chance) and excess GWG (Figure 2, Table 7, and Additional file 1: Figure S3). None of the included studies examined motivation in relation to excess GWG.

#### Psychological factors and weight gain as a secondary outcome

The findings pertaining to GWG as a secondary outcome were from a relatively small number of studies [34,39,44,47,60,64-66], and showed generally inconclusive results (Additional file 1: Table S4).

#### Cognitions related to physical activity and excess GWG (post hoc objective)

None of the included studies examined cognition related to physical activity and excess GWG.

## Discussion

This is the first systematic review to our knowledge to examine the relation between psychological predictors and excess GWG, a condition now affecting approximately half of all pregnant women in some populations. Taken together, the available evidence from 35 studies indicates that excess GWG is not related to negative affective states (such as non-clinical depression or anxiety), but is related to a number of weight-related and dietary-related cognitions, while personality traits and motivations remain underexplored constructs in relation to GWG. Specifically, negative cognitions/attitudes or inaccurate perceptions about weight gain appear to act as risk factors for excess GWG in some instances, whereas positive cognitions/attitudes appear to play a protective role. Risk factors for excess GWG may include higher levels of dietary restraint, perceived barriers to healthy eating, negative attitude towards weight gain, negative body image, concern about weight gain, high target weight gain, inaccurate perceptions regarding one’s own body weight, and less knowledge about weight gain. In contrast, protective factors include higher self-efficacy for healthy eating, lower than recommended target weight gain, and an internal locus of control with respect to weight gain. A number of these findings warrant further discussion. Overall, the evidence was more consistent in showing a lack of relation for affective states and excess GWG. However, many of the studies investigating cognitions related to weight gain were underpowered.

### Affect and excess GWG

The available evidence on negative affective states, such as symptoms of depression, anxiety and stress, indicated that they are not directly related to excess GWG. These findings are in contrast to the evidence from the general population which clearly indicates that depression and anxiety are associated with weight [67-70] with a dose–response relation between depression and weight demonstrated in a meta-analysis of 15 longitudinal studies [67] and a similar graded relation between anxiety and weight gain [69,70]. Reasons for why a relation between negative affect and excess GWG may have failed to emerge in our systematic review include firstly, the mild degree of the affective symptoms experienced by the participants included in this systematic review. This was supported by the fact that one study in our systematic review did find an association between an item pertaining to severe symptoms of depression (i.e., history of suicidal thoughts or attempts) and excess GWG [45]. A second but related reason stems from our decision to exclude studies which focused on participants diagnosed with an eating disorder. Meta-analytic evidence indicates that negative affect is a risk factor for eating pathology [71]. However, a history of severe morbidities that impact weight gain such as anorexia and bulimia together constitute less than 1% of pregnant women [72]. A third reason may be the relatively short time frame of studies during pregnancy (i.e., often only a few months), compared to the longitudinal studies in non-pregnant populations which have examined the relation between affective symptoms and weight gain across several years [67,69,70]. A growing body of research has identified ‘pregnancy-specific anxiety’ as a particularly robust risk factor for a number of negative birth outcomes [73]. It is possible that the relation between excess GWG and affect may be different for women who enter pregnancy with more negative affect compared to those whose negative affect is pregnancy-specific.

The available evidence from six of seven included studies indicates that overall, perceived stress is not related to excess GWG [2,9,36,51,56,58]. This finding was robust, given that most of the scales had been validated for use during pregnancy, two studies were large and population-based, and four studies included women from diverse ethnic backgrounds. In contrast, among non-pregnant populations, a consistent body of evidence demonstrates that higher levels of stress are associated with increased weight gain [74-76], possibly due to activation of the HPA axis, with higher glucocorticoid levels leading to increased adiposity [77,78]. Pregnancy is also associated with increased HPA axis function beginning as early as the 11^th^ week of gestation [79] and by the third trimester, blood cortisol levels are more than twofold higher among pregnant women compared to non-pregnant controls [79]. Hence there may be a ‘ceiling effect’ since levels of cortisol are higher during pregnancy regardless of the degree of stress. Hence, pregnancy-related changes in HPA axis function may dampen the relation between stress and weight gain seen in the general population.

### Cognitions related to weight gain and excess GWG

In contrast to affective states, a number of significant findings were found among studies examining negative cognitions related to weight gain and excess GWG, including most studies on *negative* attitudes towards weight gain [40,51,53,54], and negative body image (i.e., ‘thinner body size preference’) [35,46]. Furthermore, women who inaccurately perceived their weight to be greater than it was, were more likely to gain excess weight [41] as were women who were more concerned about weight gain [54] or had less knowledge about weight gain during pregnancy [54]. Consistent with the above findings, research in non-pregnant women has also shown an association between BMI tertile and negative body image [80].

Conflicting results were found between weight locus of control and weight gain during pregnancy. Consistent with the literature among non-pregnant women, which suggests that women with an internal locus of control perform better in weight loss programs [81], one underpowered study found that having an internal locus of control was associated with a lower risk of excess GWG [59]. However, the other study did not find a significant effect between either an internal or external locus of control and GWG [49], hence more research is required.

A lower than recommended target weight gain was a protective factor for excess GWG while a higher than recommended target weight gain was a risk factor for excess GWG [37,61]. The latter factor is particularly concerning given that only 29% of pregnant women reported that they were counseled to gain a certain amount of weight, but only 12%, reported receiving a recommendation in accordance with the IOM guidelines [82]. Barriers to GWG counseling by the health providers, include insufficient training, concern about the sensitivity of the topic, and the perception that counseling is ineffective [83]. Although few women reported receiving accurate recommendations about GWG and the lack of knowledge associated with excess GWG are both concerning, it is reassuring that appropriate GWG targets are a protective factor, as the provision of such information is an easily modifiable factor. This is consistent with evidence that the provision of information regarding weight gain represents a key intervention strategy in preventing excess GWG [84].

### Cognitions related to dietary behaviors and excess GWG

Although the existing evidence on cognitions related to dietary behaviors and excess GWG is limited, the available studies suggest that having higher dietary restraint, weight concerns and perceived barriers to healthy eating are all risk factors for excess GWG, while higher self-efficacy for healthy eating was a protective factor in one study [59]. Such findings are consistent with qualitative research which suggests that pregnant women regard themselves as less restrained in their eating behavior [85]. Furthermore, previous research has found that chronic dietary restraint eventually breaks down and results in impulsive eating, binge eating [86], although no studies were found investigating the association between impulsivity and GWG. Laraia [43] found that the association of restrained eating with excess GWG holds true only among women who experienced marginal food insecurity. Food insecurity appears to be associated with intake of low cost, high caloric food , which in turns is associated with weight gain [87].

The perception of barriers to healthy eating was associated with excess GWG [59], similar to the non-pregnant literature [88]. Although, no relation emerged between lack of knowledge about nutrition and weight gain among low income women [59], given that outside of pregnancy it has been perceived to be linked to weight loss in overweight and obese low income women [88], more studies are needed in a more varied pregnant population.

Self-efficacy regarding healthy eating, which in the general population is related to weight loss [89], was protective against excess GWG in one of three studies [59] but not in the others. Self-efficacy, a major domain associated with health behavior [90], may be especially important during pregnancy, a time of physiological challenges to the maintenance of health behaviors related to GWG (e.g. nausea and food cravings) [1,91]. In light of documented pregnancy-specific challenges and the modifiable nature of self-efficacy, future studies should explore whether targeting self-efficacy represents a viable intervention strategy for helping prevent excess GWG.

### Personality and excess GWG

The single study [61] which examined the relation between excess GWG and personality and found that per unit increase on Eysenck’s Neurotic Scale of Emotional Instability, the adjusted OR for excess gain was 1.26 (1.10 to 1.44). Hence, personality traits represent important areas for future research, given that outside of pregnancy a recent meta-analysis found that certain personality traits, such as conscientiousness (in an adjusted analysis) and higher openness to experience (in an unadjusted analysis), were related to a reduction in the risk of developing obesity [25], while weight fluctuation over time was predicted by personality traits such as neuroticism and impulsivity [92].

This is the first systematic review to our knowledge to examine the relation between psychological factors and excess GWG. Strengths include the comprehensiveness of the searches that were performed, using eight databases and variations on a wide selection of search terms, and the use of rigorous methodology in accordance with PRISMA (Preferred Reporting Items for Systematic Reviews and Meta-Analyses) [29]. There were a number of strengths in the methodology and design of the included studies. For example, the majority of studies investigating our primary outcomes obtained women’s final pregnancy weight from medical records, rather than self-report. Most studies were longitudinal cohort designs, allowing for stronger conclusions to be drawn regarding the predictive utility of the constructs under review and most adjusted for pre-pregnancy BMI, a known confounder of GWG [1].

The challenges associated with the current systematic review stem primarily from the limitations inherent in the individual included studies. Firstly, by considering only the relation between each construct and GWG, studies failed to evaluate a likely interaction between various psychological constructs and its impact on weight gain. AbuSabha and Achterberg [90] suggest that evaluating such constructs individually might result in weak predictive abilities and contradictory results, and this appears to have been the case so far. Secondly, the methodological quality of the included studies was not consistently high and the results should be interpreted with caution due to poor quality of some of the included studies. For instance, a large number of studies did not report effect size and confidence intervals. Moreover, many of the included studies were underpowered, and underpowered studies contribute biased effect size estimates. Thirdly, studies failed to assess whether the timings of assessment of psychological constructs during gestation had an effect on the outcome. There is a need for more work on psychological predictors of GWG according to each trimester. Fourthly, publication bias [93] may be a limitation of this systematic review but could not be assessed even with a funnel plot. Only few constructs had a sufficient number of related studies, and when such was the case (e.g., with stress), the relevant studies did not always provide sufficient information to calculate effect estimates needed to produce funnel plot, hence, limiting our ability to draw definite conclusions regarding publication bias. Fifthly, all but one of the psychological constructs assessed were not pregnancy-specific. Lastly, the considerable heterogeneity in the scales used to assess each psychological construct and the failure of many studies to report comprehensive data precluded us from computing a pooled effect estimate with meta-analytic techniques.

## Conclusion

This is the first systematic review to our knowledge to examine the relation between excess GWG and psychological antecedents, answering the call from other systematic reviews of generally non-successful interventions for preventing excess GWG to study the antecedents. Based on the studies included in this review, affective symptomatology was unrelated to excess GWG, except for severe symptoms of depression (i.e., suicidality). Negative cognitions/attitudes or inaccurate perceptions about weight gain emerged as risk factors for excess GWG whereas positive cognitions/attitudes appear to play a protective role. Specifically, risk factors for excess GWG include higher levels of cognitive dietary restraint, perceived barriers to healthy eating, negative attitude towards weight gain, negative body image, being concerned about weight gain, high target weight gain, inaccurate perceptions regarding one’s own body weight, and less knowledgeable about weight gain. Although fewer factors were protective, some evidence emerged for the association between a reduced risk of excess GWG and higher self-efficacy for healthy eating, lower than recommended target weight gain, and an internal locus of control with respect to weight gain. Finally, we identified important areas for future study, as there was little available information on the association of excess GWG with personality traits and motivation. This study forms the basis of a better understanding of psychological factors, a critical first step in developing effective interventions to prevent the current epidemic of excess GWG and help prevent the resultant trans-generational cycle of obesity. Although pregnancy spans only a short period of time, but represents a potentially critical window influencing the mother’s weight trajectory, and the fetus’ in utero programming, there is a need for further investigation on psychological factors influencing GWG.

## Additional files

Additional file 1:
**Supporting information: Final search strategies.** **Table S1.** Description of psychological scales used in the included studies. **Table S2.** Quality assessment of included cohort studies using the modified Newcastle-Ottawa scale in systematic review of psychological antecedents of excess gestational weight gain. **0 S3.** Quality assessment of included case–control and cross-sectional studies using the modified Newcastle-Ottawa scale in systematic review of psychological antecedents of excess gestational weight gain. **Figure S1.** Forest plots showing relation of affect and excess gestational weight gain in systematic review of psychological antecedents of excess gestational weight. **Figure S2.** Forest plots showing relation of cognitions related to weight gain and other cognitions and excess gestational weight gain in systematic review of psychological antecedents of excess gestational weight gain. **Figure S3.** Forest plots showing relation of cognitions related to dietary behavior and excess gestational weight gain in systematic review of psychological antecedents of excess gestational weight gain. **Table S4.** Summary table of secondary outcomes and the relation of affect, cognition related to on dietary behavior or weight gain, in systematic review of psychological antecedents of excess gestational weight gain.
