# Supplementary material for: Psychological antecedents of excess gestational weight gain: a systematic review
Source: BMC Pregnancy Childbirth. 2015 May 2;15:107. doi: 10.1186/s12884-015-0535-y (PMC4518609; doi:10.1186/s12884-015-0535-y)
Supplement: Additional file 1: — Supporting information: Final search strategies. Table S1. Description of psychological scales used in the included studies. Table S2. Quality assessment of included cohort studies using the modified Newcastle-Ottawa scale in systematic review of psychological antecedents of excess gestational weight gain. 0 S3. Quality assessment of included case–control and cross-sectional studies using the modified Newcastle-Ottawa scale in systematic review of psychological antecedents of excess gestational weight gain. Figure S1. Forest plots showing relation of affect and excess gestational weight gain in systematic review of psychological antecedents of excess gestational weight. Figure S2. Forest plots showing relation of cognitions related to weight gain and other cognitions and excess gestational weight gain in systematic review of psychological antecedents of excess gestational weight gain. Figure S3. Forest plots showing relation of cognitions related to dietary behavior and excess gestational weight gain in systematic review of psychological antecedents of excess gestational weight gain. Table S4. Summary table of secondary outcomes and the relation of affect, cognition related to on dietary behavior or weight gain, in systematic review of psychological antecedents of excess gestational weight gain. [file 12884_2015_535_MOESM1_ESM.doc]

Additional file 1 **Supporting information: Final search strategies**

Database: Ovid MEDLINE(R) In-Process & Other Non-Indexed Citations and Ovid MEDLINE(R)
--------------------------------------------------------------------------------
1     exp Pregnancy/ (711586)
2     pregnan:.mp. (782037)
3     Pregnant Women/ (5192)
4     gestation:.mp. (179760)
5     maternal.mp. (219616)
6     gravid:.mp. (18377)
7     or/1-6 (911752)
8     Weight Gain/ (22668)
9     (weight adj4 gain:).mp. (58939)
10     (weight adj4 chang:).mp. (19744)
11     (weight adj4 increas:).mp. (37899)
12     (weight adj4 excess:).mp. (6099)
13     Body Mass Index/ (79634)
14     or/8-13 (177159)
15     7 and 14 (20021)
16     stress:.mp. (632562)
17     psychologic:.mp. (352433)
18     16 and 17 (109900)
19     Stress, Psychological/ (86317)
20     distress.mp. (84671)
21     Adaptation, Psychological/ (73990)
22     (coping adj4 behavio?r).mp. (1216)
23     Self Efficacy/ (11878)
24     self efficacy.mp. (18904)
25     Anxiety/px [Psychology] (16140)
26     Depression/px [Psychology] (21065)
27     Body Image/ (12732)
28     (body adj4 image).mp. (15755)
29     Self Concept/ (44894)
30     self esteem.mp. (13320)
31     Internal-External Control/ (16163)
32     locus of control.mp. (5475)
33     Food Habits/px [Psychology] (1779)
34     Eating/px [Psychology] (1476)
35     (Eating adj4 behavio?r).mp. (4338)
36     Attitude/ (39032)
37     Attitude.mp. (228517)
38     restraint.mp. (19989)
39     knowledge/ (6810)
40     Impulsive Behavior/ (4981)
41     impulsiv:.mp. (13291)
42     Resilience, Psychological/ (1321)
43     resilienc:.mp. (8448)
44     Affect/ (24614)
45     mood:.mp. (55231)
46     Emotions/ (42087)
47     emotion:.mp. (130003)
48     Motivation/ (49879)
49     motivat:.mp. (107928)
50     Personality/ (29979)
51     "Extraversion (Psychology)"/ (2027)
52     extraver:.mp. (4089)
53     neurotic:.mp. (22550)
54     psychotic:.mp. (48315)
55     openness:.mp. (3159)
56     conscient:.mp. (2726)
57     agreeabl:.mp. (1587)
58    lie.mp. (21824)
59     erotic:.mp. (2334)
60     big five.mp. (816)
61     five factor model.mp. (847)
62     eysenck.mp. (1298)
63     or/19-62 (859887)
64     15 and 63 (1246)
65     Animal/ (5439164)
66     Human/ (13505466)
67     65 not 66 (3926196)
68     64 not 67 (1046)
***************************

**Database: Embase**

--------------------------------------------------------------------------------

1 exp pregnancy/ (616254)

2 pregnan:.mp. (776757)

3 pregnant woman/ (26808)

4 gestation:.mp. (210146)

5 maternal.mp. (243299)

6 expectant mother/ (118)

7 gravid:.mp. (21754)

8 or/1-7 (944308)

9 weight gain/ (60144)

10 (weight adj4 gain:).mp. (88406)

11 (weight adj4 chang:).mp. (25920)

12 (weight adj4 increas:).mp. (45881)

13 (weight adj4 excess:).mp. (8228)

14 Body Mass/ (166970)

15 or/9-14 (298173)

16 8 and 15 (27961)

17 stress:.mp. (786221)

18 psychologic:.mp. (702752)

19 17 and 18 (83424)

20 Stress/ (102069)

21 distress.mp. (125342)

22 anxiety/ (111722)

23 depressive symptoms.mp. (29594)

24 coping behavior/ (31298)

25 (coping adj4 behavio?r).mp. (31972)

26 self concept/ (58532)

27 (self adj efficacy).mp. (14439)

28 self esteem/ (13331)

29 self esteem.mp. (21643)

30 body image/ (14321)

31 (body adj4 image).mp. (17113)

32 "locus of control"/ (476)

33 locus of control.mp. (6045)

34 attitude/ (54145)

35 attitude.mp. (333510)

36 feeding behavior/ or eating/ or binge eating disorder/ or eating habit/ (88001)

37 (eating adj4 behavio?r).mp. (6177)

38 restraint.mp. (16879)

39 knowledge/ (17414)

40 impulsiveness/ (11135)

41 impulsiv:.mp. (17764)

42 adaptive behavior/ (47593)

43 resilienc:.mp. (9341)

44 exp affect/ (42626)

45 mood/ (23216)

46 mood.mp. (83489)

47 emotion/ (67152)

48 emotion:.mp. (182996)

49 motivation/ (67296)

50 motivat:.mp. (122452)

51 personality/ (45942)

52 extraver:.mp. or extraversion/ (5235)

53 psychotic:.mp. (36355)

54 neurotic:.mp. (15543)

55 openness:.mp. (3700)

56 conscient:.mp. (3236)

57 agreeabl:.mp. (1891)

58 lie.mp. (23085)

59 erotic:.mp. (2209)

60 Five Factor Model.mp. (1006)

61 Eysenck.mp. (1905)

62 Big Five.mp. (954)

63 or/19-62 (1293058)

64 16 and 63 (3072)

65 Animal/ (1884033)

66 Human/ (14729185)

67 65 not 66 (1403613)

68 64 not 67 (2643)

***************************

**Database: PsycINFO**

**--------------------------------------------------------------------------------**

1 exp Pregnancy/ (16494)

2 pregnan:.mp. (32582)

3 gestation:.mp. (8114)

4 maternal.mp. (39512)

5 Expectant Mothers/ (509)

6 gravid:.mp. (556)

7 or/1-6 (68620)

8 Weight Gain/ (1488)

9 (weight adj4 gain:).mp. (7841)

10 (weight adj4 change).mp. (1639)

11 (weight adj4 increas:).mp. (3168)

12 (weight adj4 excess:).mp. (775)

13 Body Mass Index/ (2209)

14 or/8-13 (13200)

15 7 and 14 (1233)

16 stress:.mp. (188915)

17 psychologic:.mp. (284352)

18 16 and 17 (39999)

19 exp Stress/ (75458)

20 Self Efficacy/ (13678)

21 self efficacy.mp. (24919)

22 Anxiety/ (41742)

23 "Depression (Emotion)"/ (21015)

24 Body Image/ (7575)

25 (body adj4 image).mp. (12153)

26 Self Esteem/ (20361)

27 self esteem.mp. (39791)

28 Internal External Locus of Control/ (12370)

29 locus of control.mp. (17398)

30 Health Attitudes/ (7873)

31 Eating Behavior/ (6726)

32 (eating adj4 behavio?r).mp. (9909)

33 restraint.mp. (8537)

34 exp Health Knowledge/ (5065)

35 Impulsiveness/ (5466)

36 impulsiv:.mp. (17177)

37 Distress/ (14214)

38 Distress.mp. (42841)

39 "resilience (psychological)"/ (6097)

40 resilienc:.mp. (13272)

41 Emotions/ (29362)

42 emotion:.mp. (280091)

43 emotional states/ (27995)

44 Motivation/ (34840)

45 motivat:.mp. (121431)

46 Personality Traits/ (39368)

47 neurotic:.mp. or Neuroticism/ (26212)

48 extraver:.mp. or Extraversion/ (10920)

49 psychotic:.mp. or Psychoticism/ (35734)

50 Openness to Experience/ or openness.mp. (8303)

51 conscient:.mp. or Conscientiousness/ (5552)

52 agreeabl:.mp. or Agreeableness/ (4047)

53 lie.mp. (8255)

54 exp Eroticism/ or erotic:.mp. (4607)

55 exp Five Factor Personality Model/ (2206)

56 exp Eysenck Personality Inventory/ (549)

57 or/18-56 (692728)

58 15 and 57 (293)

***************************

**Database: EBM Reviews - Cochrane Central Register of Controlled Trials**

--------------------------------------------------------------------------------

1 exp Pregnancy/ (14021)

2 pregnan:.mp. (19336)

3 Pregnant Women/ (56)

4 gestation:.mp. (6571)

5 maternal.mp. (6125)

6 gravid:.mp. (364)

7 or/1-6 (24170)

8 Weight Gain/ (1311)

9 (weight adj4 gain:).mp. (4097)

10 (weight adj4 chang:).mp. (2849)

11 (weight adj4 increas:).mp. (2506)

12 (weight adj4 excess:).mp. (283)

13 Body Mass Index/ (4882)

14 or/8-13 (11963)

15 7 and 14 (832)

16 stress:.mp. (18390)

17 psychologic:.mp. (16305)

18 16 and 17 (4229)

19 Stress, Psychological/ (2889)

20 distress.mp. (5373)

21 Adaptation, Psychological/ (2550)

22 (coping adj4 behavio?r).mp. (334)

23 Self Efficacy/ (1257)

24 self efficacy.mp. (2540)

25 Anxiety/ (4139)

26 Depression/ (4213)

27 Body Image/ (385)

28 (body adj4 image).mp. (618)

29 Self Concept/ (1484)

30 self esteem.mp. (934)

31 Internal-External Control/ (710)

32 locus of control.mp. (360)

33 Feeding Behavior/ (667)

34 (Eating adj4 behavio?r).mp. (377)

35 Attitude/ (758)

36 Attitude.mp. (5650)

37 restraint.mp. (513)

38 knowledge/ (91)

39 Impulsive Behavior/ (225)

40 impulsiv:.mp. (666)

41 Resilience, Psychological/ (29)

42 resilienc:.mp. (188)

43 Affect/ (3007)

44 mood:.mp. (6935)

45 Emotions/ (1677)

46 emotion:.mp. (6050)

47 Motivation/ (2565)

48 motivat:.mp. (5185)

49 Personality/ (490)

50 "Extraversion (Psychology)"/ (80)

51 extraver:.mp. (184)

52 neurotic:.mp. (847)

53 psychotic:.mp. (2509)

54 openness:.mp. (64)

55 conscient:.mp. (58)

56 agreeabl:.mp. (62)

57 lie.mp. (246)

58 erotic:.mp. (159)

59 big five.mp. (5)

60 five factor model.mp. (23)

61 eysenck.mp. (74)

62 or/18-61 (43109)

63 15 and 62 (66)

64 Animal/ (7058)

65 Human/ (4)

66 64 not 65 (7058)

67 63 not 66 (64)

***************************

**CINHAL**

--------------------------------------------------------------------------------
S48 S32 AND S47 (n=384)

S47 S15 OR S18 OR S19 OR S20 OR S21 OR S22 OR S23 OR S24 OR S25 OR S26 OR S27 OR S28 OR S29 OR S33 OR S34 OR S35 OR S36 OR S37 OR S38 OR S39 OR S40 OR S41 OR S42 OR S43 OR S44 OR S45 OR S46 (205,880)

S46 (MH "Adaptation, Psychological")

S45 "restrain*"

S44 (MH "Personality")

S43 "motivat*"

S42 (MH "Motivation")

S41 "mood"

S40 (MH "Affect")

S39 "emotion*"

S38 (MH "Emotions")

S37 (MH "Knowledge")

S36 (MH "Attitude") OR "attitude"

S35 "distress"

S34 "resilience"

S33 "impulsiv*"

S32 S8 AND S31 (n=3616)

S31 S9 OR S10 OR S11 OR S12 OR S13 OR S14 OR S30 (n=36,472)

S30 (MH "Body Mass Index")

S29 (MH "Eating Behavior")

S28 (MH "Depression/PF")

S27 (MH "Anxiety/PF")

S26 body N4 image

S25 (MH "Body Image")

S24 locus of control

S23 (MH "Locus of Control")

S22 self esteem

S21 (MH "Self Concept")

S20 self efficacy

S19 (MH "Self-Efficacy")

S18 S16 AND S17

S17 psychologic*

S16 stress

S15 (MH "Stress, Psychological")

S14 (MH "Body Weight Changes")

S13 weight N4 increas*

S12 weight N4 access*

S11 weight N4 chang*

S10 weight N4 gain*

S9 (MH "Weight Gain")

S8 S1 OR S2 OR S3 OR S4 OR S5 OR S6 OR S7

S7 maternal

S6 gravid*

S5 gestation*

S4 childbearing or child bearing

S3 (MH "Expectant Mothers")

S2 pregnan*

S1 (MH "Pregnancy")

******************************************************************************

**Web of Knowledge**

--------------------------------------------------------------------------------
# 1 297,751

Topic=(pregnan*)

# 2 113,093

Topic=(gestation*)

# 3 156,429

Topic=(maternal)

# 4 8,108

Topic=(gravid*)

# 5 443,733

#4 OR #3 OR #2 OR #1

# 6 123,527

TS=(Body Mass Index) or TS=(BMI)

# 7 54,716

TS=(Weight Near/4 Gain*)

# 8 969

Topic=(Weight Near/4 access*)

# 9 53,630

Topic=(Weight Near/4 increas*)

# 10 26,154

Topic=(Weight Near/4 chang*)

# 11 232,976

#10 OR #9 OR #8 OR #7 OR #6

# 12 17,683

#11 AND #5

# 13 30,568

Topic=(stress) AND Topic=(psychologic*)

# 14 22,550

Topic=("self efficacy")

# 15 22,433

Topic=("self esteem")

# 16 5,345

Topic=("locus of control")

# 17 15,450

Topic=(Body Near/4 Image)

# 18 28,715

Topic=(impulsiv*)

# 19 89,335

Topic=(distress)

# 20 23,773

TS=(resilienc*)

# 21 62,568

Topic=(mood)

# 22 146,795

TS=(emotion*)

# 23 177,268

Topic=(motivat*)

# 24 176,200

Topic=(attitude)

# 25 1,444

TS=(eat* Near/4 behavio?r)

# 26 13,111

Topic=(coping behavior)

# 27 20,078

Topic=(personality trait*)

# 28 725,743

#27 OR #26 OR #25 OR #24 OR #23 OR #22 OR #21 OR #20 OR #19 OR #18 OR #17 OR #16 OR #15 OR #14 OR #13

# 29 849

#28 AND #12

******************************************************************************

**Sociological abstract-3 May 2013 (N=9)**

((SU.EXACT("Pregnancy") OR SU.EXACT("Maternal/Maternity") OR if(gravid*) OR if(pregnan*) OR if(gestation*)) AND (SU.EXACT("Body Weight") OR if(weight NEAR/4 excess) OR if(weight NEAR/4 gain*) OR if(weight NEAR/4 chang*) OR if(weight NEAR/4 increas*))) AND (SU.exact("KNOWLEDGE") OR su.Exact("psychological stress" OR "psychological distress") OR (psychologic* AND stress) OR (su.Exact("self esteem") OR (self esteem)) OR (su.Exact("selfefficacy") OR (self NEAR/4 efficacy)) OR (su.Exact("locus of control") OR (locus of control)) OR (su.Exact("body image") OR (body NEAR/4 image)) OR su.Exact("anxiety-depression") OR attitude OR (su.Exact("impulse/impulsiveness/ impulsivity") OR impulsiv*) OR (su.Exact("resilience") OR resilienc*) OR (su.Exact("mood" OR "affect") OR mood*) OR (su.Exact("emotion") OR emotion*) OR (su.Exact("motivation") OR motivat*) OR (su.Exact("adapt/adaptive/adaptability/ adaptation") OR (coping behavio?r)) OR (su.Exact("personality") OR personality) OR (extravert*) OR (neurotic*) OR (psychotic*) OR (openness*) OR (conscient*) OR (agreeabl*) OR (lie*) OR (erotic*) OR (big five*) or (five factor model) OR (eysenck) OR (su.Exact("eating behaviour") OR (eating behavio?r)))

**Dissertation and thesis**

(all(((su.Exact("stress" OR "self esteem") OR diskw(self NEAR/4 efficacy OR locus of control OR body NEAR/4 image OR anxiety OR depression)))) OR su.Exact("attitudes") OR su.Exact("knowledge") OR su.Exact("personality traits") OR all(distress) OR all(impulsiv*) OR all(resilienc*) OR (su.Exact("motivation") OR all(motivat*)) OR (su(food habit) OR all((diet* habit* OR diet* behavio?r)) OR all(feeding behavio?r)) OR (su.Exact("emotions") OR all(emotion*) OR all(mood)) OR (extravert*) OR (neurotic*) OR (psychotic*) OR (openness*) OR (conscient*) OR (agreeabl*) OR (lie*) OR (erotic*) OR (big five*) or (five factor model) OR (eysenck) OR (su.Exact("adaptation") OR all(coping behavio?r) OR all(adapt*))) AND ((su(pregnancy) OR diskw(pregnan*) OR diskw(gravid*) OR diskw(gestation*) OR diskw(maternal)) AND (diskw(weight NEAR/4 gain*) OR diskw(weight NEAR/4 increas*) OR diskw(weight NEAR/4 excess*) OR diskw(weight NEAR/4 chang*)))

**Table S1: Description of psychological scales used in the included studies**

| **Construct assessed** | **Scale used (original scale / model reference)** | **Description** |
| --- | --- | --- |
| Anxiety | State and Trait Anxiety Inventory (STAI) | Twenty item scale; responses on a 4-point Likert-type scale; contains two subscales: State and Trait; Higher state anxiety scores reflect greater current anxious affect, whereas higher Trait anxiety scores indicate a more general tendency to experience elevated levels of anxiety. |
|  | Depression, Anxiety, and Stress Scale-21 (DASS-21) | Twenty-one item scale; 7 questions to measure each of three negative emotional states of depression, anxiety, and stress; responses on a 4-point Likert-type scale; low scores reflect less severe states |
| Attitude towards weight gain | Pregnancy and weight gain attitude scale | Eighteen item scale rated using a 5-point Likert scale ranging from 1 (strongly disagree) to 5 (strongly agree). Low scores represent a more negative attitude towards weight gain in pregnancy. |
|  | Kendall | Thirteen item questionnaire; 5-point Likert scale ranging from very unimportant to very important |
|  | Investigator developed (Strychar 2000), based on Theory of Planned Behavior | Twenty-six items divided into eight sub-scales: intention to gain recommended weight; favorable or unfavorable attitude; salient beliefs; behavioral beliefs; subjective norms; normative beliefs; motivation to comply; and perceived behavioral control |
| Barriers to Health Eating | Fowles' Barriers to Health Eating Scale (BHES) | Eleven items ascertaining women’s environmental and internal difficulties with respect to buying and preparing healthy food |
| Pre-pregnancy body image dissatisfaction | Body Image Assessment for Obesity (BIA-O) | Eighteen silhouettes ranging from very light to very obese body sizes, on 18 cards. To identify Current Body Size (CBS), the cards are shown to the participants in a random order and they are asked to select the silhouette that was similar to their pre-pregnancy body size. The number of the selected card (1 to 18) is recorded as the CBS score. To determine Rational Body Size (RBS), the cards are reshuffled and participants are asked to select the silhouette that they perceive as the sensible body size for their pre-gravid stage. The number of this card is recorded as their RBS score. A body size dissatisfaction score was calculated for each participant by subtracting the RBS score from the CBS score. This score was then used to classify participants into three categories: dissatisfied with a heavier body size preference, satisfied, or dissatisfied with a thinner body size preference |
|  | Body Attitudes Questionnaire | Twenty-eight items divided into 4 subscales: feeling fat (12 items); strength and fitness (6 items); salience of weight and shape (5 items); and attractiveness: (5 items). |
|  | *Body Area Satisfaction Scale | Nine items, 5-point Likert scale ranging from 1 (very dissatisfied) to 5 (very satisfied); Participants were asked to rate their degree of body satisfaction with specified body parts such as thighs, face, or stomach as well as their height, weight, and muscle tone |
|  | Stunkard  Figure  Rating  Scale and validation to BMI | Nine line figures); Participants were asked to rate their perception; misestimation of BMI =( discrepancy score (desired-actual) line figure |
| Cognitive dietary restraint | Revised Restraint Scale | Ten items divided into two subscales i.e., Weight Fluctuation (or cyclers) and Concern with Dieting (or dieters) |
|  | Dutch Eating Behaviour  Questionnaire (DEBQ) | Based on the DEBQ, participants were classified as emotional, external, or retrained eaters. Emotional eating was measured with 13 items, four items measured diffuse emtions and nin specific emotions. External and restrained eating were measured by 10 items each. |
| Concerns and beliefs about weight gain | Investigator developed, based on Health Belief Model | Fourteen items divided into 4 subscales: perceived concern, risk, benefits, and barriers |
| Coping strategy | Psychological Acculturation scale | Ten items, encompassing four dimensions (cultural loyalty, solidarity, comprehension, and identification) are used to assess psychological responses to cultural exposure among Peurto-Ricans in the USA. The 5-point Likert scale ranged from 1 (only Hispanic/Latino orientation) to 5 (only Anglo-American orientation), with a bicultural orientation at the mid-point. |
|  | *Coping strategy scale | Twelve item scale with 3 subscales on Personal Influence; Acceptance; Social Assurance |
| Depression | Centre for Epidemiological Studies-Depression Scale (CES-D) | Twenty items, 4-point Likert scale with regard to symptoms during the past week. Four subscales are: depressive affect (7 items), somatic (7 items), well-being (4 items), and interpersonal difficulties (2 items). |
|  | Edinburgh Postnatal Depression Scale (EPDS) | Ten items, 4-point Likert scale, with overall scores ranging from 0 to 30. Validated antenatal cut-points exist for "probable major depression" (an overall score ≥15) and for "at least probable minor depression" (an overall score ≥13 |
|  | Patient Health Questionnaire (PHQ) | Eight items on a 4-point Likert scale (0 to 3 points); Assessed in regard to symptoms during the past two weeks; Current depressive symptoms were defined as a score ≥ 10 |
|  | Depression, Anxiety, and Stress Scale-21 (DASS-21) | (see Anxiety) |
| Eating disorder | Eating Disorder Examination Questionnaire (EDEQ) | Twenty-eight items, 6-point Likert scale. A section of the questionnaire measuring disordered eating to was used to assess objective overeating, binge eating and purging behavior |
| Eating habits | *Eating Habits subscale of the Weight and Life Style Inventory (WALI) | Twenty-four items on a 5-point Likert scale; 1 (does not contribute at all) to 5 (contributes the greatest amount); Areas covered were overeating at meals, snacking between meals, snacking after dinner, eating due to physical hunger, eating due to cravings, and eating when anxious, bored, stressed, angry, depressed/upset, or alone |
|  | *Night Eating Questionnaire (NES) | Fourteen item questionnaire assesses the behavioral and psychological symptoms of NES. The scale measures two core features of NES, evening hyperphagia and nocturnal ingestions, along with associated features; A cut score above 30 is strongly suggestive of night eating syndrome. |
| Fetal Health Locus of Control | Fetal Health Locus of Control scale (FHLC) | Eighteen items with three subscales: Internality, Powerful Others and Chances. Scores are calculated for each component with higher scores reflecting stronger beliefs. |
| Knowledge about nutrition | Investigator developed | Twenty four items scale based on USA dietary recommendations and culturally appropriate pictures of portions of food. |
| Self-esteem | Self-esteem scale | Ten item scale rated on a 6-point Likert scale to evaluate participants’ general elimination of self-worth and degree of orientation (positive or negative) towards the self. Higher scores reflect more positive self-appraisals. |
|  | Single-item Self-Esteem Scale (SISE) Robins | Single-item scale; 5-point Likert scale “I have high self-esteem”. Higher scores reflect more positive self-appraisals. |
| Self-efficacy | Self-efficacy scale | Eight items: two items reflecting control of food intake, two items on confidence about getting regular exercise after pregnancy, two items about confidence to return to pre-pregnancy weight and shape, and one on food intake |
|  | Self-efficacy for healthy eating based on theory of planned behavior and Bandura’s theory of perceived self-efficacy | Five items with two options for each item: 1) intention/motivation to manage weight during pregnancy and 2) ability to prepare healthy meals and cook with vegetables. |
| Stress | Perceived Stress Scale (PSS) | Fourteen items on a 5-point Likert scale ranging from 0 (never) to 4 (almost always). After reverse scoring positively worded items, a total score is calculated by summing across all items; Higher scores reflect greater distress and lower confidence in personal coping resources; A short form perceived stress scale has four items |
|  | Modified Life Event Inventory | Checklist of 59 life events which represent the relative severity of psychosocial stresses; Each item is scored on a 1 to 100 scale according to the amount of “turmoil, disturbance, or upheaval” it would cause were it to happen; PRAMS questionnaire contains an 18-item subset of the Modified Life Events Inventory |
|  | Prenatal Life Events checklist  designed on the basis of the Life Events scale | 19-item scale classiﬁed into 4 domains, including ﬁnancial, emotional, traumatic,and spouse-related domains with 2 additional items left blank for additional factors; dichotomous (yes/no) response to indicate whether the event had occurred; to recall when it had occurred, by which the gestational age was determined; and to weight their perception of its impact on their emotional wellbeing from no impact (0) to extreme impact (4); the scores were summed, A total score of 0, 1, or 2 or greater was deﬁned as, respectively, no stress, modest stress, or severe stress |
|  | Prenatal Psychosocial Profile Hassles Scale | Eleven items on a 4-point Likert format ranging from ''no stress'' to ''severe stress''; the scale measures pregnancy-specific daily hassles and uplifts |
|  | Depression, Anxiety, and Stress Scale-21 (DASS-21) | (see Anxiety) |
| Weight Locus of Control | Weight Locus of Control scale | Four items indicative of whether a person believes she has control over body weight (internal locus of control) or whether weight is something over which she has little control (external locus of control) |
| Weight concerns | Dieting and Binge Eating Severity Scale (DBESS) | Six subscales where non-dieters and casual dieters are classified as low weight concern; intense dieters, severe dieters, at-risk dieters and probable bulimic classified as high weight concern |

*scale used in secondary outcome studies only; PRAMS: Pregnancy Risk Assessment Monitoring System

**References for Table S3**

**Table S2: Quality assessment of included cohort studies using the modified Newcastle-Ottawa scale in systematic review of psychological antecedents of excess gestational weight gain**

| **Author Year** | **Selection** | | | **Comparability of cohorts**  (maximum:**) | **Outcome** | | | **Overall score (out of 8)** |
| --- | --- | --- | --- | --- | --- | --- | --- | --- |
|  | Representativeness of exposed cohort  (maximum:*) | Selection of non-exposed cohort  (maximum:*) | Ascertainment of exposure-validated tools  (maximum:*) |  | Assessment of outcome  (maximum:*) | Sufficient follow-up duration  (maximum:*) | Adequate follow-up  (maximum:*) |  |
| Allison 2012 | - | * | * | ** | * | * | - | ******  (6) |
| Brawarsky 2005 | * | * | * | ** | * | * | * | ********  (8) |
| Chasan-Taber 2008 | * | * | * | ** | * | * | - | *******  (7) |
| Cogswell 1999 | * | * | - | ** | - | * | - | *****  (5) |
| Copper 1995 | - | * | * | ** | * | * | - | ******  (6) |
| Herring 2008 | * | * | - | ** | * | * | - | ******  (6) |
| Hill 2013 | - | * | * | ** | - | * | - | *****  (5) |
| Laraia 2013 | * | * | * | ** | * | * | - | ********  (7) |
| Loris 1985 | - | - | - | - | * | * | - | **  (2) |
| McAnarney 1992 | * | * | * | - | * | * | * | ******  (6) |
| McPhie 2015 | * | * | * | ** | - | - | - | *****  (5) |
| Mehta 2011 | * | * | * | ** | * | * | * | ********  (8) |
| Mehta-Lee 2013 | - | * | - | ** | * | * | * | ******  (6) |
| Morling 2003 | * | * | - | ** | - | * | - | *****  (5) |
| Mumford 2008 | * | * | * | ** | * | * | - | *******  (7) |
| Olson 2003 | * | * | * | * | * | * | * | *******  (7) |
| Pomerleau 2000 ^$^ | - | * | * | * | - | - | - | ***  (3) |
| Stevens-Simon 1993 | - | * | * | * | * | * | - | *****  (5) |
| Stevens-Simon 1995 | * | * | * | - | * | * | * | ******  (6) |
| Strychar 2000^$^ | - | * | - | ** | - | * | - | ****  (4) |
| Sui 2013 | - | * | - | * | * | - | - | *****  (4) |
| Tovar 2012 | * | * | * | ** | * | * | - | *******  (7) |
| van der Wijden 2014 | - | * | * | ** | * | - | - | *****  (5) |
| Walker 2002 ^$^ | * | * | * | ** | - | * | - | ******  (6) |
| Webb 2009 | * | * | * | ** | * | * | - | *******  (7) |
| Wells 2006 | - | * | - | * | - | * | - | ***  (3) |
| Wright 2013 ^$^ | - | * | - | ** | - | * | * | *****  (5) |
| Zhu 2013 | - | * | - | ** | * | * | - | *****  (5) |
| Zuckerman 1989 | * | * | * | * | * | - | * | ******  (6) |

Legend: Ascertainment of exposure-validated tools= ‘≥ 50 % of the tool(s) are stated /known validated’ or ‘≥ 50 % of the tool(s) are validated, but modified; Comparability= 1* for controlled for pre-pregnancy BMI and studies were given 1 additional * if they controlled for one or more of the following five potential confounders: age, parity, income, education or race; Assessment of outcome required objective measurement of GWG not self-report; Sufficient duration of follow-up for total GWG required evaluation at 37 weeks or beyond; adequate follow-up defined as >90% follow-up ^$^ Cross-sectional study, however, treated similar to a cohort study by authors; -did not qualify to receive a score for a particular criterion

**Table S3: Quality assessment of included case-control and cross-sectional studies using the modified Newcastle-Ottawa scale in systematic review of psychological antecedents of excess gestational weight gain**

| **Author Year** | **Selection** | | | **Comparability of cases and controls**  (maximum:**) | **Exposure** | | | **Overall score (out of 8)** |
| --- | --- | --- | --- | --- | --- | --- | --- | --- |
|  | Adequate case definition  (maximum:*) | Representativeness of cases  (maximum:*) | Selection of controls  (maximum:*) |  | Assessment of exposure  (maximum:*) | Same method of ascertainment for cases and controls  (maximum:*) | Non-response rate  (maximum:*) |  |
| Bagheri 2013 | * | - | * | ** | * | * | - | ******  (6) |
| Conway 1999^$^ | - | - | * | - | * | * | * | ****  (4) |
| Dipietro 2003 ^$^ | - | - | * | * | * | * | - | ****  (4) |
| McDonald 2013^$^ | - | - | * | ** | * | * | - | *****  (5) |
| Sangi-Haghpeykar 2013 ^$^ | - | - | * | ** | * | * | - | *****  (5) |
| Walker 2009 | * | * | * | ** | - | * | - | ******  (6) |

Legend: Assessment of exposure= ‘≥ 50 % of the tool(s) are stated /known validated’ or ‘≥ 50 % of the tool(s) are validated, but modified’; ^$^Cross-sectional study, however, treated similar to a case-control study by authors; -did not qualify to receive a score for a particular criterion

**Figure S1: Forest plots showing relation of affect and excess gestational weight gain in systematic review of psychological antecedents of excess gestational weight gain**
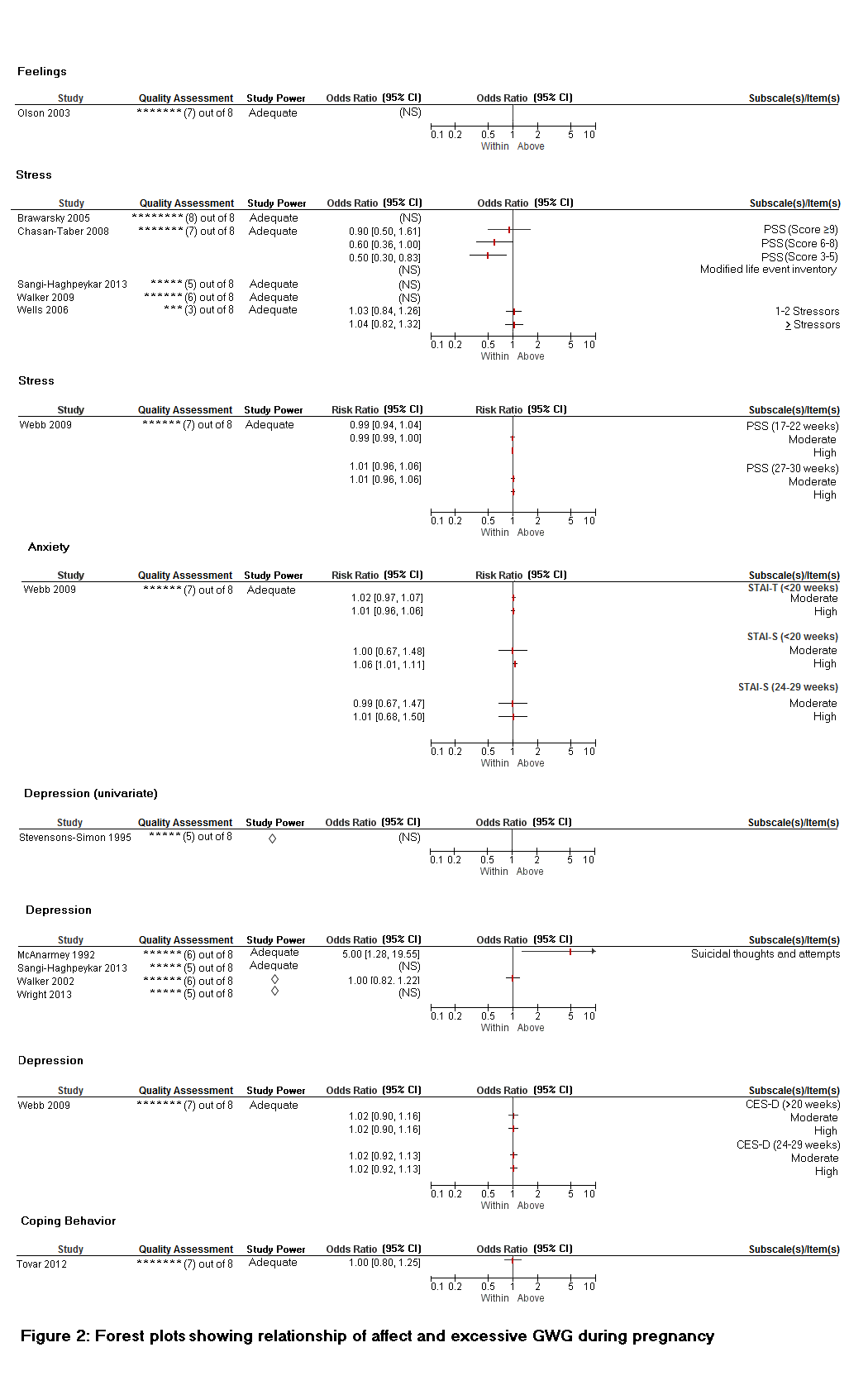


**Figure S2: Forest plots showing relation of cognitions related to weight gain and other cognitions and excess gestational weight gain in systematic review of psychological antecedents of excess gestational weight gain**


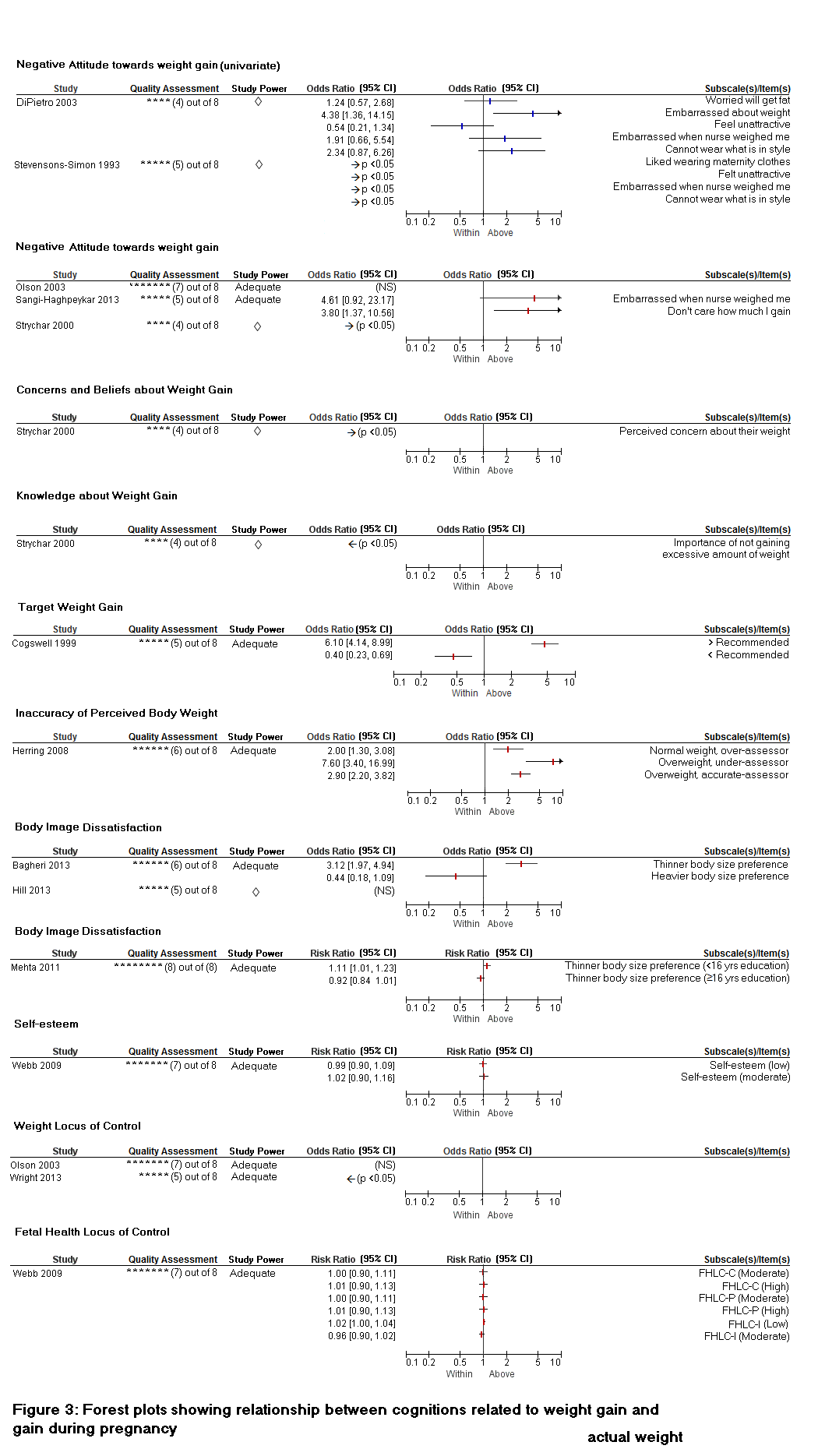


**Figure S3: Forest plots showing relation of cognitions related to dietary behavior and excess gestational weight gain in systematic review of psychological antecedents of excess gestational weight gain**


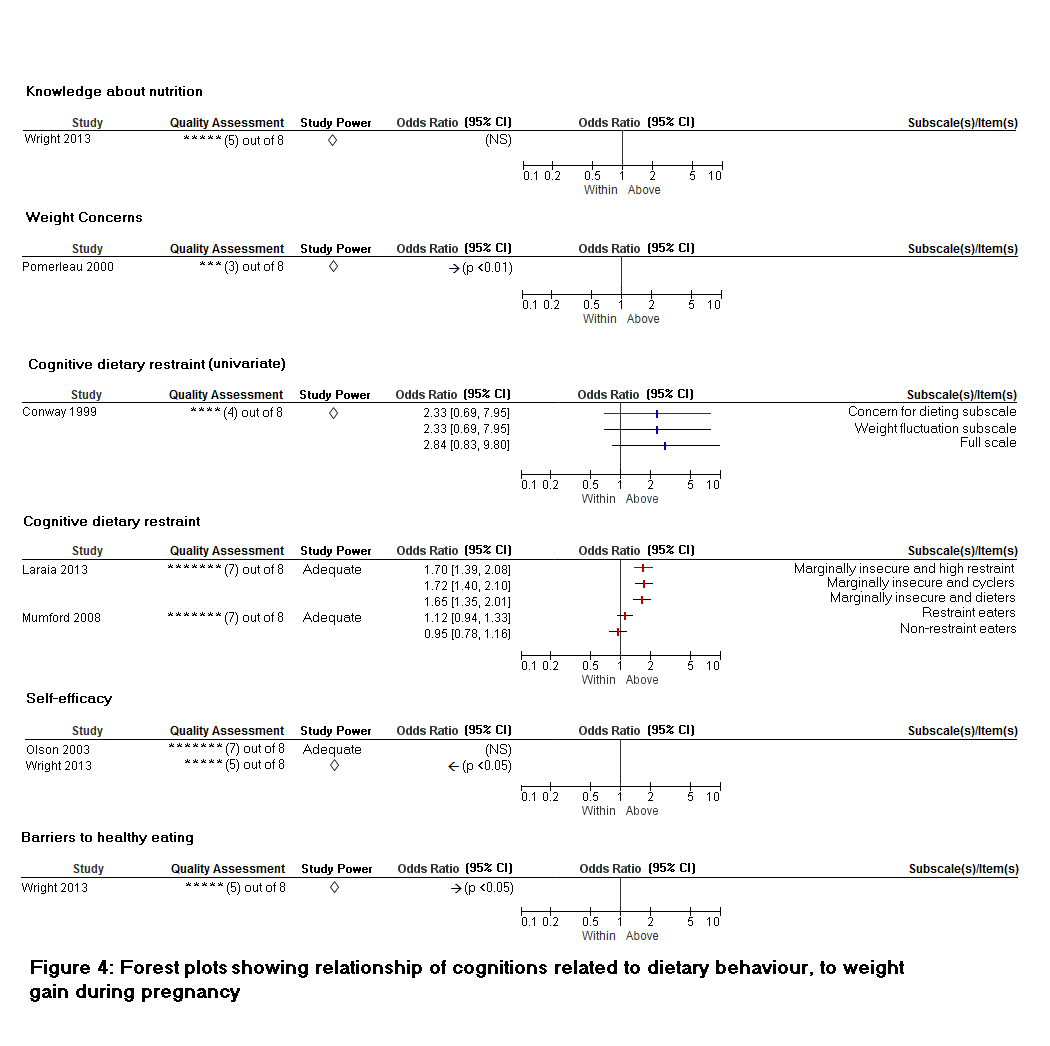


**Legends for Figure S1 – S3**

Number of * = quality scoring of the study according to the modified NOS; = under powered; 🡪= Positive association (risk factor); 🡨 = Negative association (protective factor); NS = not significant; the box and the bars represent the effect estimate and 95% *CI*, respectively for each study that presented this data for which it could be calculated; CES-D = Centre for Epidemiological Studies-Depression Scale; FHLC-C = Fetal Health Locus of Control -Chances Scale; FHLC-I = Fetal Health Locus of Control -Internality Scale; FHLC-P = Fetal Health Locus of Control -Powerful Others Scale; Multivariate analysis is presented unless specified in the titles of the forest plot as a univariate analysis; PHQ = Patient Health Questionnaire; PSS = Perceived Stress Scale; STAI-S = State and Trait Anxiety Inventory-State; STAI-T = State and Trait Anxiety Inventory-Trait. Forest plots report multivariate analyses, unless labeled in the title as univariate.

**Table S4: Summary table of secondary outcomes and the relation of affect, cognition related to on dietary behavior or weight gain, in systematic review of psychological antecedents of excess gestational weight gain**

| **Author** | **Scale used*** | **Outcome for univariate** | **Crude (unadjusted) results** | **Adjusted results** | **Confounders adjusted for** | **Summary of results** |
| --- | --- | --- | --- | --- | --- | --- |
| **Exposure: Feeling** | | | | | | |
| Loris 1985 | Investigator developed, single item on feeling towards pregnancy, Not validated | GWG^$$^ | Mean (±SD):  Feeling categorised as:  Great: 42.4 (17.0)  OK or Good: 35.0 (15.5) | NA | NA | NS on univariate analysis; multivariate analysis not done |
| **Exposure: Depression** | | | | | | |
| Allison 2012 | EPDS, Validated | GWG | Correlation co-efficient (p-value):  r=0.039 (p-value NS) | NA | NA | NS on univariate analysis; variable not entered in a multivariate model |
| Zuckerman 1989 | CES-D, Validated | Gestational Weight Gain (in lb) categorised as  <=10 lb;11-20lb; 21-30lb; >=31lb  No reference to pre-pregnancy BMI | Mean CES-D score:  ≤ 10lb 22.2  11-20lb 19.1  21-30lb 18.4  ≥ 31lb 17.6 | NA | NA | 🡨Significant  on univariate analysis; multivariate analysis was not done |
| **Exposure: Stress** | | | | | | |
| Zhu 2013 | Stressful Life Events scale, not reported as being validated | GWG (continuous) | Retrospectively assessed stress in 1^st^ trimester at 32 weeks, Β coefficient of stressful life event ( 95% CI): -0.564 (-0.890 to -0.238);  Retrospectively assessed stress in 2nd trimester at 32 weeks, Β coefficient of stressful life event ( 95% CI): −0.199 −0.571 to 0.172); 3^rd^ trimester -0.333 (-0.772 to 0.105) | Retrospectively assessed stress in 1^st^ trimester at 32 weeks, Β coefficient of stressful life event ( 95% CI): -0.497 (-0.817 to -0.176); 2^nd^ trimester −0.137 (−0.503 to 0.228); 3^rd^ trimester -0.312 (-0.744 to 0.120) | maternal age, education attainment, income, newborn gender, gestational age at delivery, and pre-pregnancy BMI | Significant on univariate and multivariate analysis  🡨 (Significant in retrospective assessment of 1^st^ trimester, but not 2^nd^) |
| **Exposure: Coping strategy** | | | | | | |
| Morling 2013 | Investigator developed, Coping Strategy Scale, Not Validated | GWG | NR | Effect estimates not reported for composite score; (p-value NS)  Interaction between Acceptance (item 4) with country (β ± S.E.):  American -1.94 (±0.57)  Japanese 0.75 (±0.54) | Pre-pregnancy weight, parity, weight gained and intended to gain during pregnancy as reported in trimester 1 | Only an interaction between 1 item and country (American) was significant on multivariate analysis |
| **Exposure: Negative attitude towards weight gain** | | | | | | |
| Copper, 1995 | Pregnancy and weight gain attitude scale, Validated | GWG | Correlation co-efficient (p-value):  r =0.05 (p =0.08) | Effect estimate not reported; (p <0.001) | Pre-pregnancy BMI, infant sex, race, tobacco use, and gestational age at delivery | Significant on multivariate analysis  🡨 |
| van der Wijden 2014 | Pregnancy and Weight  Gain Attitude Scale, validated scale | GWG (continuous) | NR | B coefficient (95 % CI) of healthy pregnancy attitude:  assessed at 15 weeks, -0.80 (-2.37, 0.77)  B coefficient (95 % CI) of weight gain attitude:  assessed at 15 weeks, 0.36 (-0.90, 1.62) | BMI, age and the time between the two  measurements of weight (~15-35 weeks) | NS in the multivariate model |
| **Exposure:** Self-efficacy | | | | | | |
| Sui 2013 | Self-efficacy, not validated | GWG (continuous) | Effect estimate not reported (non-significant) | NA | NA | NS |
| van der Wijden 2014 | Self-efficacy, validated scale | GWG (continuous) | NR | B coefficient (95 % CI):  assessed at 15 weeks, 1.25 (-0.08, 2.59) | BMI, age and the time between the two  measurements of weight (~15-35 weeks) | NS in the multivariate model |
| **Exposure: Eating habits** | | | | | | |
| Allison 2012 | Weight and Life Style Inventory (WALI), Validated | Excess GWG for univariate;  GWG (continuous) for multivariate | Correlation co-efficient (p-value)  Items from a subscale:  Eating due to craving  r=0.21 (p <0.05);  All other items were NS | β (95% CI ):  4.7 (0.6 to 8.7) | Pre-pregnancy BMI, age, gestational age, education, smoking | Only 1 item was significant on multivariate analysis  🡪 |
| Allison 2012 | Night Eating Questionnaire (NEQ), Validated | GWG | Correlation co-efficient (p-value):  r=0.053 (p-value NS) | NA | NA | NS on univariate analysis; variable not entered in multivariate analysis |
| **Exposure: Cognitive dietary restraint** | | | | | | |
| Allison 2012 | Three Factor Eating Questionnaire (item number 51), Validated | GWG | Correlation co-efficient:  r=-0.023 (p-value NS) | NA | NA | NS on univariate analysis; variable not entered in a multivariate model |
| **Exposure: Inaccurate estimation of BMI** | | | | | | |
| Sui 2013 | Stunkard Figure Rating Scale, not stated if validated | GWG (continuous) | Misestimation of BMI associated with higher GWG (i.e. correlation between  ( discrepancy score (desired-actual) and GWG  (R  2  = 0.133, P = 0.018), indicating that women with a high degree of  body  image dissatisfaction were more likely to have higher  GWG | NA | NA | Significant on univariate analysis; not assessed with multivariate |
| **Exposure: Eating disorder** | | | | | | |
| Allison 2012 | Eating Disorder Examination Questionnaire (EDEQ) , Validated | GWG | Correlation co-efficient:  Sub-item:  Overeating episodes  r=0.057 (p-value NS)  Binge episodes  r=0.104 (p-value NS) | NA | NA | NS on univariate or multivariate analyses |
| **Exposure:** Restrained eating | | | | | | |
| van der Wijden 2014 | Dutch Eating Behavior Questionnaire. Validated | Excess GWG (continuous) | B coefficient (95 % CI):  Restrained eating assessed at 15 weeks, 1.39 (0.08, )2.71; (emotional eating not reported at 15 weeks) | B coefficient (95 % CI):  Restrained eating assessed at 15 weeks, 0.09 (-0.91, 1.08);  Emotional eating assessed at 15 weeks, 0.22 (-0.83, 1.25) | BMI, age and the time between the two  measurements of weight (~15-35 weeks) | Restrained eating significant on on univariate 🡪;  NS in the multivariate model  Emotional eating NS in the multivariate |

*Scale details can be found in Supplementary Table S1; ^$^2009 IOM GWG guidelines; ^$$^ GWG measured in pounds (lb); 🡪Positive association (Risk factor); 🡨 Negative association (Protective factor); GWG: Gestational Weight Gain; NA: Not Applicable; NR: Not Reported; NS: Not Significant
